# Cryptic kin discrimination during communal lactation in mice favours cooperation between relatives

**DOI:** 10.1038/s42003-023-05115-3

**Published:** 2023-07-15

**Authors:** Jonathan P. Green, Catarina Franco, Amanda J. Davidson, Vicki Lee, Paula Stockley, Robert J. Beynon, Jane L. Hurst

**Affiliations:** 1 Mammalian Behaviour & Evolution Group, Institute of Infection, Veterinary and Ecological Sciences, University of Liverpool, Leahurst Campus, Neston, CH64 7TE UK; 2 Centre for Proteome Research, Institute of Systems, Molecular and Integrative Biology, University of Liverpool, Crown Street, Liverpool, L69 7ZB UK; 3 Present Address: Department of Biology, University of Oxford, 11a Mansfield Road, Oxford, OX1 3SZ UK; 4 Present Address: MRC Laboratory of Molecular Biology, Francis Crick Avenue, Cambridge Biomedical Campus, Cambridge, CB2 0QH UK

**Keywords:** Social evolution, Animal behaviour

## Abstract

Breeding females can cooperate by rearing their offspring communally, sharing synergistic benefits of offspring care but risking exploitation by partners. In lactating mammals, communal rearing occurs mostly among close relatives. Inclusive fitness theory predicts enhanced cooperation between related partners and greater willingness to compensate for any partner under-investment, while females are less likely to bias investment towards own offspring. We use a dual isotopic tracer approach to track individual milk allocation when familiar pairs of sisters or unrelated house mice reared offspring communally. Closely related pairs show lower energy demand and pups experience better access to non-maternal milk. Lactational investment is more skewed between sister partners but females pay greater energetic costs per own offspring reared with an unrelated partner. The choice of close kin as cooperative partners is strongly favoured by these direct as well as indirect benefits, providing a driver to maintain female kin groups for communal breeding.

## Abstract (web summary)

A dual isotope tracer approach assessed milk allocation when pairs of sisters or unrelated female house mice reared offspring communally, revealing that females pay greater energetic costs when rearing offspring with an unrelated partner.

## Introduction

Cooperative behaviour, where individuals provide assistance to others despite costs to their own reproduction, plays a central role in the evolution of sociality. Much attention has focused on cooperative breeding, when breeders are helped by other group members that sacrifice or delay their own reproduction, or else help when own breeding has failed^1–3^. Such costly cooperation has evolved predominantly in kin groups^4–6^, where helpers can gain indirect fitness benefits from rearing young that carry a proportion of their genes^7^ when harsh ecological conditions favour helping strategies^8,9^, or social grouping at high density aids resource defence^10^. Nonetheless, in some systems, subordinates can gain sufficient direct benefits, through group living and improved survival or territory inheritance, for helping of unrelated breeders to evolve^11,12^. By contrast, communal breeders (when two or more females in the same group breed at the same time) can cooperate by rearing their offspring communally; they may share synergistic benefits of provisioning, defence and other aspects of offspring care, while each gains reproductive success^13–17^. However, individuals are vulnerable to exploitation in communal breeding systems if partners fail to invest proportionally in the communal brood^18–20^, or if their offspring vary in ability to gain the synergistic benefits available (for example, younger offspring may have lower ability to compete for resources)^14,21^. Across taxa, communal rearing of young occurs among both related^13,14,22–24^ and unrelated females^16,25–28^. For unrelated females, enhanced direct fitness to all parties can maintain cooperation in the absence of shared genetic interests^25–27^. When communal rearing occurs within kin groups, which is particularly common in mammals, this may reflect enhanced fitness benefits of cooperating with kin, but may alternatively be a consequence of existing kin structure within populations^29,30^.

A crucial decision facing communally-breeding females is how much to invest in the brood, and particularly to own offspring versus the offspring of other breeders. Inclusive fitness theory predicts that kinship between females should affect this decision in two ways. First, investment in care of another female’s young should be reduced when co-breeding females are unrelated, since no indirect fitness benefits are available when helping non-relatives. However, females would need to target any reduced investment effectively to avoid harming own offspring, which may be hard to achieve in many communal nesting scenarios where offspring are fully mixed and compete for resources^31^. Second, females will be more likely to compensate for underinvestment (exploitation) by related partners, since they stand to gain indirect benefits if lightening the load of a related partner improves that partner’s lifetime fitness^17,32,33^. Because of this greater willingness to compensate, females that pay higher lifetime fitness costs for the same level of investment as their partner (for example, those in poorer condition) are predicted to be more likely to underinvest when cooperating with a related partner to improve their lifetime reproductive fitness. Related partners should be willing to compensate rather than abandon the communal litter if the cost of this additional required investment to their direct fitness does not exceed their likely fitness gains from rearing both own and related offspring. Together, this predicts a greater bias in investment towards own offspring when females breed communally with unrelated partners, but greater skew in total investment between closely related breeding partners. Further, if females favour kin partners and experience increased efficiency and/or reduced harm when cooperating with kin, these benefits may translate into greater direct fitness through enhanced number, survival and/or quality of offspring when females choose close kin as cooperative breeding partners.

Studies to date have compared the productivity of communal breeding groups comprising either related or unrelated females, finding that reproductive success is greater when mothers are related^18,32,34–36^. However, tracking individual investment in different offspring within communal broods to understand how relatedness between partners and offspring influences investment is very challenging. This is particularly the case in mammals where lactational investment is cryptic and observation of suckling behaviour is not a reliable measure of investment^37,38^. Here, we develop an approach to solve this problem, using stable isotope labelling to accurately track investment made by each partner mother in each offspring in a communal litter as well as measuring the energy intake of each partner female. We then apply this to pairs of female wild-stock house mice rearing offspring communally to address: i) whether there is any bias in investment provided to own and other offspring; whether kinship between partners alters ii) the amount that females invest, iii) the extent of investment bias between partners; and iv) any difference in direct benefits gained by individual females when cooperating with related or unrelated partners.

Female house mice live in family-based territorial social groups and facultatively raise offspring either in solitary nests or communally with familiar partners (most usually in pairs) that share the same nest sites^30,39^. When both opportunities are available, choice appears to be condition- and density-dependent, with younger females choosing communal rearing most frequently while communal rearing increases at high density^40,41^. Improved survival of litters appears to be the main benefit of communal compared to solitary rearing, due to the vulnerability of newborn pups to infanticide from other conspecifics, particularly when mothers are absent from the nest^32,36,42–45^. Older and more experienced females can rear more pups per surviving litter when rearing solitarily compared to females breeding communally, but most females in a high-density free-living house mouse population were only able to rear any surviving offspring by cooperative communal rearing^40,41^. Relatives are strongly preferred as nest partners, discriminated through similarity of odours^46,47^. However, females will rear offspring communally with tolerated non-relatives^32,35,36,48,49^. Under laboratory conditions, where females can be compared under identical circumstances, lifetime reproductive success (measured as the number and biomass of own offspring produced by females over a 6 month lifetime) is higher when cooperating with a littermate sister than with an unrelated female, or when rearing offspring alone^34,42^. While observational data show that communally breeding females appear not to discriminate and spend similar time with own and partner offspring, fully mixed from birth in communal nests, it is not known whether females transfer more milk to own than to partner offspring^19,37^. Nor is it known whether offspring themselves discriminate between mothers in communal nests. Laboratory mouse pups imprint on own mother’s odour in utero and use this to find and attach to their mother’s teats when first born^50^. Changes in milk quality through lactation^51^, which pups can discriminate^52^, might further influence preferences between mothers in communal nests.

By successfully tracking individual maternal food intake and milk investment among pairs of females raising offspring communally, we show that littermate sisters raise the same number and weight of offspring as socially compatible unrelated partners, but are more efficient, requiring less energy to rear communal broods of equivalent mass. Sisters show more skewed investment in the communal brood as predicted, but both sister partners pay significantly lower energy costs per own offspring reared than females cooperating with an unrelated partner. While pups gain milk from both cooperating partners, cryptic kin discrimination in offspring provision is evident as pups gain slightly more milk from their own mother than expected from partner energy investment and younger pups are competitively disadvantaged in gaining milk from unrelated partners but not from an aunt. Our results show a strong selective advantage for the choice of close relatives as communal rearing partners due to reduced costs per own pup reared compared to cooperation between unrelated partners.

## Results

### Assessment of individual investment

The first challenge was to devise a strategy to determine accurately the investment gained by each pup from different mothers in communal nests formed by pairs of familiar close relatives or familiar but unrelated females. We used harmless stable isotopes to differentially label the diet of each mother, using two variants of the amino acid lysine: one labelled with four deuterium atoms [^2^H_4_]lysine, the other with nine deuterium atoms [^2^H_9_]lysine (hereafter referred to as d4 and d9 lysine). The labelled amino acids were each mixed into standard diet as free amino acid to double the total lysine content and give a relative isotope abundance (RIA) of 0.5 (i.e. the proportion of lysine labelled). On ingestion, the amino acid is incorporated into newly synthesized proteins, including those in milk. Labelled milk proteins ingested by pups are hydrolysed in the gut to free amino acids, absorbed and incorporated into pup tissues. The labelling of pup proteins with d4 and d9 lysine then provides a measure of the relative investment gained from two communally nursing mothers fed on diets with these different labels.

**Fig. 1:**
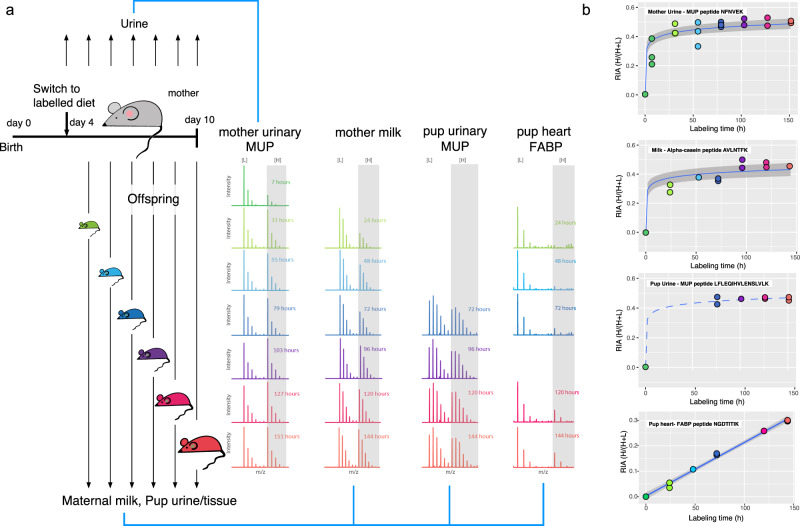
Isotope flow from mother to pup. **a** Four days after giving birth, three pairs of communally nursing female BALB/c mice were given a diet containing [^13^C_6_]lysine (diet RIA = 0.5, all females received the same diet). Proteins in daily samples of maternal urine, pup urine, milk (recovered from a pup’s stomach) and pup tissues were analysed for the incorporation of lysine isotope by LC-MS of tryptic peptides. Different peptides are shown for each source (mothers: NFNVEK from urinary MUP; milk: AVLNTFK from alpha-casein; pup urine: LFLEQIHVLENSLVLK from urinary MUP; pup heart tissue: NGDTITIK from fatty acid binding protein). As [^13^C_6_]lysine is heavier than natural lysine, labelled lysine can be distinguished by a higher mass LC-MS isotopomer profile ([H]: heavy [^13^C_6_]lysine isotope profile shaded grey; [L]: light natural lysine profile). **b** Increase in label incorporated into peptides over 7 days on labelled diet, calculated as the relative isotope abundance (RIA), i.e. the proportion of total lysine that was heavy (H/(H + L)). The blue line is a fitted first order curve with 95% confidence limits shaded in grey. For fatty acid binding protein from pup heart (FABP), the fitted curve is linear.

We conducted preliminary experiments using laboratory mice (BALB/c) to establish the suitability of the labelling strategy and optimize the measure of relative investment. Pairs of females with a communal litter were provided with [^13^C_6_]lysine in their diet and we successfully monitored the incorporation of isotope in maternal and pup proteins over a 6 day period via labelling of maternal and pup major urinary proteins (MUPs), milk and pup tissues (Fig. 1). Because the extent of labelling was subject to diurnal variation in food or milk intake^53,54^, pup urinary proteins mostly reflect recent suckling by pups. To provide a stable measure of relative female investment, we elected to use pup tissue proteins to assess the time-integrated incorporation of labelled isotopes into expanding tissue pools as pups grew. To select suitable proteins, we conducted a proteome analysis of pup heart tissue by LC-MS/MS, from which we could calculate the RIA of different proteins. As expected, the RIA differed between individual proteins, reflecting the rate of pool expansion in growing pups and the endogenous turnover rate of each protein (Supplementary Fig. 1a). Because the diet contained equal amounts of unlabelled and labelled lysine, the maximum RIA that could be reached was 0.5. After 6 days, some proteins had barely acquired any label due to their low turnover rate and little or no growth-related pool expansion. Other proteins had acquired substantial label and reached RIA values of >0.35 within one week, as expected for high turnover proteins or those undergoing pool expansion associated with pup development. As we were interested in the relative amount of milk (label) gained from two mothers nursing communally, the rate of turnover or pool expansion is not critical, except that more highly labelled proteins allow more accurate measurement of relative investment, while very high turnover proteins are vulnerable to diurnal variation in isotope intake. Thus, we based our analysis on an intermediate turnover protein, fatty acid binding protein (Uniprot P11404; Fig. 1b). Irrespective of the RIA of individual proteins, concordance between peptides from any single protein was very high (Supplementary Fig. 1b, *r* > 0.98). We also confirmed the consistency of the calculation of relative investment when pups gained milk from two differently labelled females, when different proteins were compared (Supplementary Fig. 2), attesting to the robustness of assessment of relative investment.

**Fig. 2:**
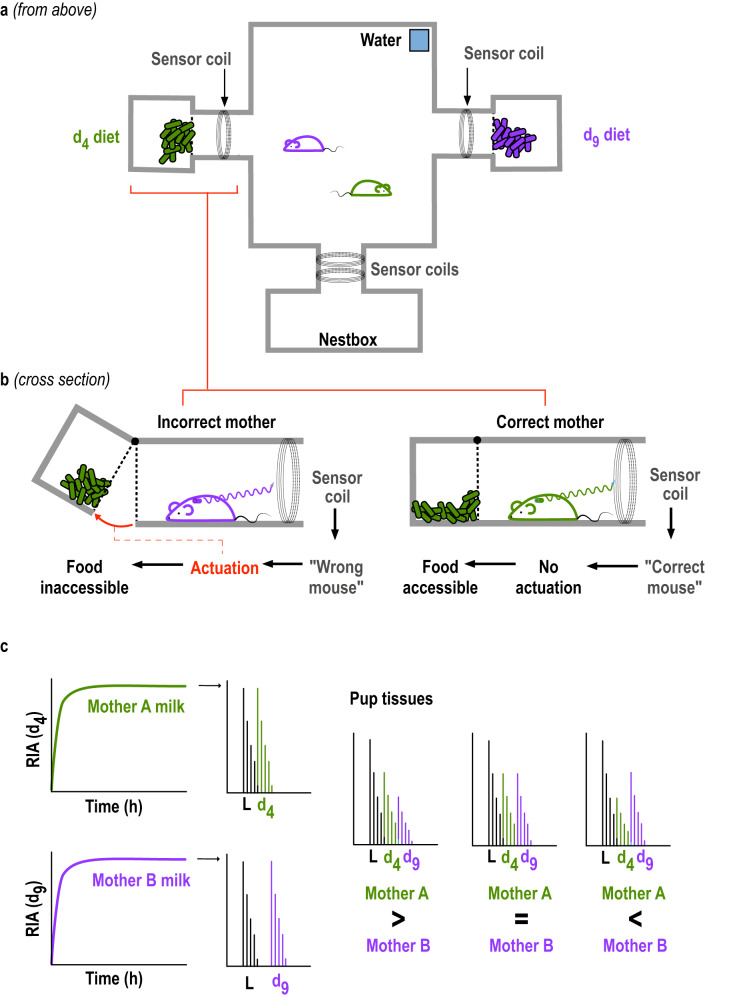
Administration of differently labelled diets to communally nursing females. **a** Cage set-up housing each pair of females. Offspring were reared in a nestbox accessed through a tunnel, with visits monitored continuously via a pair of RFID antennae. Two additional tunnels led to separate feeders containing d4 or d9 labelled pelleted diet, accessed through a metal grill. **b** RFID antenna detected females entering the feeder tunnels and moved the food away from the access grill when the mother assigned to the other labelled diet entered the tunnel. Each pair of females was trained to use the feeder system prior to breeding. **c** LC-MS analysis of urine samples from each mother confirmed that they accessed their own diet almost exclusively (Supplementary Fig. 3), while the proportion of d4 and d9 labelled lysine in pup tissue at the end of the labelling period measured the total investment gained from each mother (representations of typical isotope patterns).

Assessment of individual investment during communal rearing required females living together to each retain exclusive access to their specific labelled diet. We designed a cage system in which d4 and d9 labelled diets were provided in separate food hoppers, accessed by separate tunnels (Fig. 2a). Diet availability was controlled through a radio frequency identification (RFID) sensor system which removed access to the diet when the ‘wrong’ female entered the tunnel, detected through a subcutaneous RFID tag (Fig. 2b). Isotope analysis of serum albumin from maternal urine samples collected at the end of the labelling period (Supplementary Fig. 3) confirmed the restriction of each mother to her assigned diet, apart from a low level of labelling likely to have been acquired through dropped food crumbs or (rarely) a labelled pup that was eaten, readily corrected for during analysis (Supplementary Note 1).

Peptide labelling for pups receiving milk from both mothers was complex, with peptides incorporating d4 and d9 lysine from different mothers, together with unlabelled lysine (d0) from both mothers (Fig. 2c). As there was slight overlap between the three isotopomer profiles (due primarily to the natural abundance of 13 C, see Fig. 2c), we calculated the theoretical isotopomer distribution from the elemental composition of each peptide and used this to correct for the overlap (Supplementary Note 1).

We then applied this strategy to assess maternal investment during communal rearing in eight pairs each of littermate sisters or age-matched unrelated females. By using littermate sisters, we ensured that related partners had both the genetic (similar inherited odours) and environment cues (familiarity in utero and during shared rearing) that this species uses to detect relatedness. As female house mice choose familiar, socially compatible partners for communal rearing (whether related or unrelated)^32,48^, we cohoused each pair of females for at least one month prior to breeding to ensure a high degree of adult familiarity and social tolerance. We used only pairs that showed no aggression towards each other after their initial introduction. To control for relatedness between pups that might influence competitive interactions, we mated sisters to males that were not related to each other while unrelated females were mated to a pair of brothers (unrelated to the females). Thus, each communal brood consisted of full sibs and maternal or paternal cousins, although this design would not control for possible differences in competitive kin discrimination between maternally and paternally related pups (see Methods). Pairing with males was staggered within pairs to promote a small age difference of up to 5 days between litters, typical of communal nests in free-living mice^30^. As female house mice often lose their litters within the first one or two days after giving birth, whether breeding singly or with another female (particularly primiparous females), maternal food was labelled in communally breeding pairs for 7d once the first born litter was aged 7 days and the second born litter was 2-7 days of age. There were no differences in the relative timing of births between sister and non-sister pairs (see Methods for full details; numbers of pups per female and age difference between litters in each communal nest are shown in Supplementary Fig. 4). We assessed the total amount of milk invested by each female from their individual food intake, which rises sharply during lactation as mice are ‘income breeders’ and milk is not stored^51,55^. The total milk investment received by each pup was assessed according to pup body weight at the end of the labelling period (day 14: oldest pups aged 14 d, just prior to taking solid food), while the proportion of total milk that each pup received from each female was provided by the proportion of labelled fatty acid binding protein that was d4 or d9 (Fig. 2c). We also monitored the time that each female spent in the nest with pups via two RFID sensor coils surrounding a tunnel that led into the nest box where females constructed their communal nest (Fig. 2b; see Methods for full details).

### Female kinship reduces energy required to rear pups communally

**Fig. 3:**
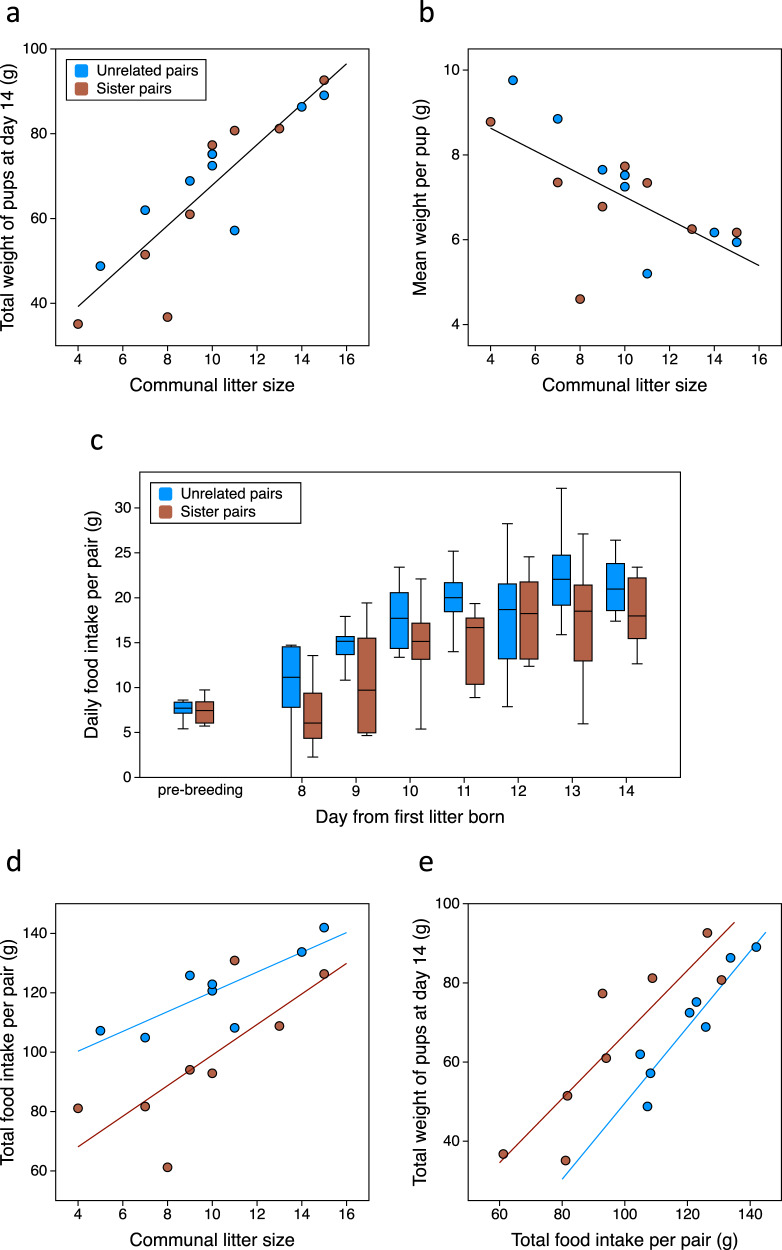
Kinship reduces energy required to rear pups in communal nests. Total weight of pups at day 14 (oldest pups 14d old) increased with communal litter size (**a**, *p* < 0.0001) but mean weight per pup reduced (**b**, *p* = 0.009), with no difference between sister (red, *n* = 8) and unrelated (blue, *n* = 8) communal nests. **c** Daily food intake in unrelated pairs (median ± IQR boxes, whiskers show full range) was higher than in sister pairs during lactation (days 8–14, *p* = 0.023) but not before breeding (pre, *p* = 0.91). Food intake per pair increased with communal litter size (*p* = 0.0001) but unrelated pairs ate more than sister pairs with the same combined number of pups (**d**, *p* = 0.016) and achieved a lower weight of pups for the amount of food eaten (**e**, *p* = 0.028). *P* values indicate significance from analyses of covariance shown in Table 1 and Supplementary Table 1. Linear regression lines shown where there was a significant relationship (separate lines for sister and unrelated pairs where these differed or combined when they did not).

Females formed communal nests in all eight sister pairs and eight unrelated pairs where both females gave birth within 5 days of each other and pups from both litters survived (see Methods for details of females that did not breed or lost their pups so could not be included in the study). The litters produced and reared communally by sisters or unrelated females were very similar. There was no difference in the number of pups born (sisters: 11.3 ± 1.1, unrelated: 10.8 ± 1.4 pups per nest [mean ± *SEM*], *F*_1,14_ = 0.08, *p* = 0.78; range 5–17) or the number of pups surviving to day 14 (sisters: 9.6 ± 1.2, unrelated: 10.1 ± 1.2 surviving per nest, *F*_1,14_ = 0.09, *p* = 0.77; range 4–15). There was also no difference in the age gap between first and second-born litters (sisters: 2.3 ± 0.5 d, unrelated: 2.5 ± 0.6 d *F*_1,14_ = 0.10, *p* = 0.75), or the mean age of pups at day 14 (sisters: 12.9 ± 0.2 d, unrelated: 12.7 ± 0.3 d, *F*_1,14_ = 0.28, *p* = 0.60). In agreement with previous studies^18,34,35^, the growth of pups was very similar in sister and unrelated communal broods (Supplementary Table 1), with no difference in the total weight of pups at day 14 (*p* = 0.90, Fig. 3a), or the mean weight achieved per pup (*p* = 0.71, Fig. 3b). As expected, total pup mass increased strongly with the number of pups in the communal nest (*p* < 0.0001, Fig. 3a). The total weight of pups also increased with the mean body weight of the two mothers prior to breeding (*p* = 0.019) and age of younger pups at day 14 (*p* = 0.013, Supplementary Table 1). However, the mean weight achieved per pup reduced as communal litter size increased (*p* = 0.009, Supplementary Table 1; Fig. 3b), confirming that female milk investment increases with demand from more pups but does not fully compensate for increasing numbers of offspring^51,55^.

While sister and unrelated pairs achieved a very similar weight of pups by day 14, we discovered that the energy investment required to rear the same weight of pups was 18% higher on average for unrelated pairs, indicating reduced costs when females cooperated with a familiar close relative. Maternal daily food intake increased substantially as pups grew across the 7 d labelling period as expected (*F*_6,84_ = 15.63, *p* < 0.0001). However, lactating sister pairs consistently ate less food per day than unrelated pairs (sisters: 14.4 ± 1.2 g per day; unrelated 17.8 ± 0.7 g per day; *F*_1,14_ = 6.49, *p* = 0.023), a difference that was not evident before breeding (*F*_1,27_ = 0.01, *p* = 0.91; Fig. 3c). Female food intake increased with communal litter size as expected (*p* = 0.0001), but sisters ate significantly less than unrelated pairs that reared the same number of pups (*p* = 0.016, Table 1; Fig. 3d). A small number of pups disappeared from nests during the 7 d labelling period (6 pups from sister pairs, 2 pups from unrelated pairs), most likely eaten when pups failed to thrive. Correction for the total number of pups present on each day of the labelling period provided a very similar but slightly tighter relationship, with sisters eating significantly less than unrelated females that had the same pup load (*p* = 0.011; Supplementary Fig. 5). Further, while the total weight of pups achieved by day 14 depended on how much food the two mothers ate (*p* < 0.0001), sisters produced a greater weight of pups for the quantity of food eaten (*p* = 0.028, Table 1; Fig. 3e).

**Table 1:** Relatedness between mothers influences the relationship between total food intake, communal litter size and pup weight.

Effect^a^	Partial *η* ^2^	*F*	df	*p*^b^
**Total food eaten by pair**				
Communal litter size at day 14	0.751	33.26	1,11	**0.0001**
Relatedness between mothers	0.421	7.99	1,11	**0.016**
Mean weight of mothers prior to breeding	0.427	8.19	1,11	**0.015**
Age of 2^nd^ born litter at day 14	0.125	1.58	1,11	0.24
**Total weight of pups at day 14**				
Total food eaten by pair	0.839	57.20	1,11	**<0.0001**
Relatedness between mothers	0.366	6.36	1,11	**0.028**
Mean weight of mothers prior to breeding	0.169	2.24	1,11	0.16
Age of 2^nd^ born litter at day 14	0.066	0.77	1,11	0.40

^a^Analysis of covariance, *n* = 16 nests (8 sister pairs, 8 unrelated pairs), data in Fig. 3 and Supplementary Data 1.

^b^Values in bolded text are statistically significant (*p* < 0.05).

Improved thermoregulation of young pups has been suggested as a potential advantage of communal rearing, reducing energy demand because pups spend less time alone in the nest than those reared by a single mother^14,56^. However, this is not likely to explain the difference in energy requirement between communally nursing sisters versus unrelated females as their pups spent a similar proportion of time alone in the nest (sisters 25.6 ± 5.0%, unrelated 23.3 ± 2.2% time when pups were alone, *F*_1,13_ = 0.20, *p* = 0.66, taking communal litter size into account; see also ref. ^49^). Counter-intuitively, the less time that females spent in the nest, the greater the weight of pups achieved, due to a tradeoff between time in the nest and time spent foraging to produce milk (Supplementary Fig. 6). This suggests that other factors are responsible for the greater energy required for communal rearing by unrelated partners, examined further below.

### Pups gain less investment from a partner mother

**Fig. 4:**
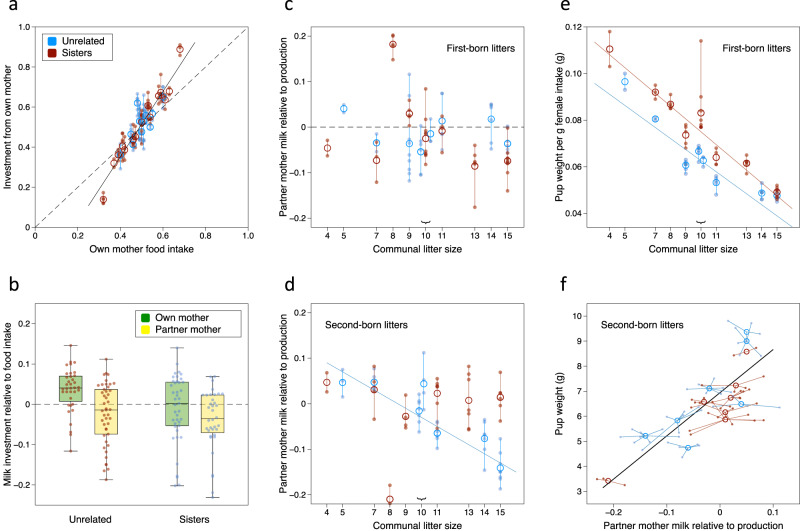
Pups gain less investment from a partner mother than expected from female food intake, leading to poorer growth among less competitive younger pups in communal nests. **a** Relative investment gained from own mother according to the mother’s food intake relative to her partner (a measure of relative milk production) by pups in sister (red) and unrelated (blue) litters (dashed line shows expected investment based on own mother intake). **b** Relative investment gained per pup from a randomly assigned focal vs other mother, corrected for the bias expected from relative female food intake (see Table 1). The randomly assigned focal mother was either the pup’s mother (green) or partner mother (yellow). Effect of communal litter size (competition) on the proportion of milk gained from the partner mother minus the proportion expected from female food intake for pups in first-born (**c**) or second-born (**d**) litters in sister (red) or unrelated (blue) pairs. Increasing competition reduced the proportion of milk gained from unrelated partner mothers in second-born litters (**d**, blue regression line). **e** In first-born litters, pups fed by sisters (red) gained more weight per g female food intake due to greater food intake among unrelated females (blue). **f** In second-born litters, pups that gained less partner milk than expected from female food intake gained less weight across both sister (red) and unrelated (blue) nests. Data are means per litter (large open circles) or median ± IQR boxes with whiskers showing full range, and individual data points (small filled circles; *n* = 44 sister and *n* = 41 unrelated pups in first-born litters; *n* = 33 sister and *n* = 40 unrelated pups in second-born litters). Corresponding statistical analyses are shown in Table 2 (**a**, **b**), Table 3 (**c**, **d**) and Table 4 (**e**, **f**). Regression lines shown where there was a significant relationship (solid lines).

Next, we examined the relative milk investment that pups gained from each female to establish whether pups gain greater investment from their own mother, and whether any bias increases when pups are not related to the cooperating partner. The food intake of each partner mother during lactation determines the energy that they invest. At a whole nest level, the proportion of label that pups gained from female A in each pair, averaged over all pups in the communal nest, correlated very strongly with the difference in food intake between female A and her partner (*r* = 0.95, *p* < 0.0001). This confirms that our surrogate measure was an accurate reflection of the proportion of energy invested by each female. At the level of individual pups, the relative difference in food intake between partners over the labelling period was the strongest predictor of the proportion of protein that pups gained from each female in both sister and unrelated communal nests (*p* < 0.0001, Table 2; Fig. 4a). Each 1% bias in food intake between partners resulted in a 1.6 ± 0.1% bias in milk gained from the mother that ate the most food. However, pups also gained a slightly greater proportion of milk from their own mother than predicted by relative maternal food intake (3.0 ± 0.1% more milk from own mother across all pups, *p* = 0.047, Table 2; Fig. 4b). This did not differ between pups in sister and unrelated nests (*p* = 0.57, Table 2).

**Table 2:** Proportion of investment gained by pups from focal female in each pair^a^.

Effect^b^	Fixed effect	χ^2^	df	*p*^c^
Own mother	0.030 ± 0.014	3.94	1	**0.047**
Female food intake^d^	1.607 ± 0.145	34.95	1	**<0.0001**
Relatedness between females	−0.013 ± 0.022	0.33	1	0.57

^a^Focal female was randomly assigned within each pair.

^b^Mixed-effects models include nest and mother as random effects. Likelihood ratio tests compared models with versus without each specific factor or interaction. Data for all pups from 16 communal nests (77 pups in sister nests, 81 pups in unrelated female nests), shown in Fig. 4 and Supplementary Data 1.

^c^Values in bolded text are statistically significant (*p* < 0.05).

^d^Proportion of food intake over the labelling period that was due to the focal female.

Greater milk gained from own mother could result from several mechanisms: (i) mothers directly bias milk delivery towards own pups; (ii) pups prefer own mother’s milk; (iii) pups lose out in competition for non-maternal milk. While the first two mechanisms predict a general bias in milk gained from own mother across all pups, reduced access to partner milk due to pup competition predicts an increased bias with greater competition (communal litter size) and among pups that are younger than their communal nest-mates because they will be competitively disadvantaged by their earlier developmental stage and smaller size^14^. To investigate further, we looked at the effects of communal litter size and litter birth-order on the relative investment that pups gained from the partner mother, taking into account pup relatedness to the partner female as well as the partner’s relative milk production (measured as proportion of total food eaten by the pair). This revealed a significant interaction between all four factors (*p* = 0.043; Table 3). This was because competition differentially influenced access to a partner mother’s milk among younger (less competitive) pups depending on their relatedness to the partner mother, while competition did not affect access by older pups in the nest. Pups in first-born litters (0-5 days older than their littermates) gained milk from the partner mother in proportion to the partner’s relative milk production (food intake); this was not influenced by competition between pups (communal litter size) or by relatedness to the partner mother (Table 3; Fig. 4c). By contrast, competitive access to partner mother milk by pups in second-born litters differed between sister and unrelated female nests (interaction between communal litter size, relatedness and partner’s relative food intake, *p* = 0.008, Table 3). Pups in second-born litters that were related to the partner mother gained milk in proportion to the partner’s relative milk production (food intake) regardless of the level of competition (Table 3; Fig. 4d: red symbols). However, when unrelated to the partner mother, the proportion of milk that pups gained from the partner reduced with increasing competition (communal litter size, *p* = 0.0003, Table 3; Fig. 4d: blue symbols). When competition for milk was high (more pups than the 10 teats of a single mother), unrelated second-litter pups gained 10 ± 1% less milk from the partner mother than predicted by her relative food intake (range 4–19% less). Thus, bias in maternal investment in unrelated nests arose largely because the younger, second-born pups were less competitive at gaining milk from an unrelated female.

**Table 3:** Effect of communal litter size and litter birth order on relative investment gained from partner mother.

Effect	Fixed effect	χ^2^	df	*p*^a^
***All pups***^b^				
Communal litter size	−0.006 ± 0.003	5.83	1	**0.016**
Pup from 1^st^ vs 2^nd^ born litter	−0.035 ± 0.017	4.09	1	**0.043**
Partner food intake^c^	1.675 ± 0.110	68.24	1	**<0.0001**
Partner relatedness	−0.001 ± 0.016	0.01	1	0.94
4 way interaction		20.18	11	**0.043**
Relatedness × communal litter size × birth order		10.49	5	0.063
***First-born litters***^d^				
Communal litter size	−0.005 ± 0.003	2.34	1	0.13
Partner food intake	1.696 ± 0.144	38.63	1	**<0.0001**
Partner relatedness	−0.018 ± 0.020	0.81	1	0.37
***Second-born litters***^d^				
Communal litter size	−0.007 ± 0.004	3.61	1	0.057
Partner food intake	1.683 ± 0.160	31.06	1	**<0.0001**
Partner relatedness	0.020 ± 0.025	0.64	1	0.42
Relatedness × communal litter size × partner food intake		13.70	4	**0.008**
Relatedness × communal litter size		9.34	1	**0.002**
***Second-born litters in sister nests***^d^				
Communal litter size		0.14	1	0.71
Partner food intake		21.30	1	**<0.0001**
***Second-born litters in unrelated nests***^d^				
Communal litter size		12.84	1	**0.0003**
Partner food intake		11.68	1	**0.0007**

^a^Values in bolded text are statistically significant (*p* < 0.05).

^b^Mixed-effects models of relative investment gained from partner mother (mother included as a random effect in each model; pair had zero variance and so was not included). Interactions that had significant effects are shown. Data for all pups from 16 communal nests (*n* = 44 first born and *n* = 33 second born pups in sister nests; *n* = 41 first born and *n* = 40 second born pups in unrelated female nests), shown in Fig. 4 and Supplementary Data 1.

^c^Proportion of food intake over the labelling period that was due to partner mother.

^d^Analysis broken down into four different subsets to interpret the significant 4 way interaction for all pups. Adjustment for multiple post hoc comparisons using a conservative Bonferroni correction has  a threshold for significance of *p* < 0.0125.

This might have little effect on pups if they are able to compensate and gain more of the energy required to grow quickly from their own mother. Individual pup body weights at day 14 were reduced with increasing communal litter size (Fig. 3b) and increased by greater maternal food intake in both first and second born litters (Table 4). However, pups in second-born litters were disadvantaged if they gained less milk from the partner mother than expected from the female’s relative milk production, achieving lower growth (*p* = 0.0015) when other factors that also influenced pup weight were taken into account (communal litter size, total maternal food intake, pup age, own mother and partner body weights, Table 4; Fig. 4f). By contrast, the body weight of the first-born litter pups was not influenced significantly by the proportion of milk gained from the partner mother. Instead, pup sex (males gained more weight, *p* = 0.008) and relatedness between partner mothers (*p* = 0.01) influenced weight achieved by first-born litter pups when communal litter size and maternal food intake were taken into account (Table 4). First-born litter pups reared by sisters achieved a greater weight by day 14 than expected from maternal food intake and communal litter size (on average, 1 g more per pup at age 14 days; Fig. 4e). This was not because first-born litter pups achieved greater weight in sister nests, but because sisters required less food to produce the same weight of pups (see above, Fig. 3e). This relationship was not evident in second-born litters though, where access to partner milk had a substantial impact on weight achieved. This included one sister nest of eight pups where the mother of the first-born litter made little investment in communal feeding and the mother of the second-born litter provided most of the milk to both litters. While the first-born litter pups (4d older) grew at the rate expected for the communal litter size and total female food intake supported largely by their aunt (Fig. 4e), the second-litter pups gained very little from the under-investing partner sister and grew very slowly (Fig. 4f) in competition with their older nestmates for their own mother’s milk.

**Table 4:** Factors influencing the body weight achieved by pups by day 14.

Effect^a^	Fixed effect	χ^2^	df	*p*^b^
***Pups in first born litters***				
Communal litter size	−0.452 ± 0.068	21.87	1	**<0.0001**
Total female food intake	0.056 ± 0.012	14.69	1	**0.0001**
Pup sex	0.222 ± 0.077	7.96	1	**0.005**
Relatedness	0.985 ± 0.317	7.59	1	**0.006**
Proportion of partner milk adjusted^c^	0.721 ± 0.926	0.60	1	0.44
Mother weight (g)	0.006 ± 0.056	0.01	1	0.91
Partner mother weight (g)	0.081 ± 0.039	3.88	1	**0.049**
***Pups in second-born litters***				
Pup age at day 14	0.513 ± 0.077	20.36	1	**<0.0001**
Communal litter size	−0.482 ± 0.064	25.51	1	**<0.0001**
Total female food intake	0.054 ± 0.011	16.44	1	**<0.0001**
Pup sex	0.135 ± 0.086	2.37	1	0.12
Relatedness	0.415 ± 0.232	2.99	1	0.084
Proportion of partner milk adjusted^c^	3.30 ± 0.997	9.20	1	**0.002**
Mother weight (g)	0.110 ± 0.032	8.45	1	**0.004**
Partner mother weight (g)	−0.096 ± 0.044	4.21	1	**0.040**

^a^Mixed-effects models include mother as a random effect. Data for all but one male pup from 16 litters (*n* = 44 first-born and *n* = 33 second-born litter pups in sister nests; *n* = 41 first-born and *n* = 40 second-born litter pups in unrelated female nests). One male pup from unrelated nest B with an abnormally large recorded weight was excluded as a strong outlier but made no difference to conclusions (see Statistics and Reproducibility section in Methods). For first-born litters, all pups were 14 days of age at day 14 so age was not included in the model.

^b^Values in bolded text are statistically significant (*p* < 0.05).

^c^Proportion of milk gained from partner mother minus proportion of total maternal food intake.

### Individual milk investment is more skewed between sisters

**Fig. 5:**
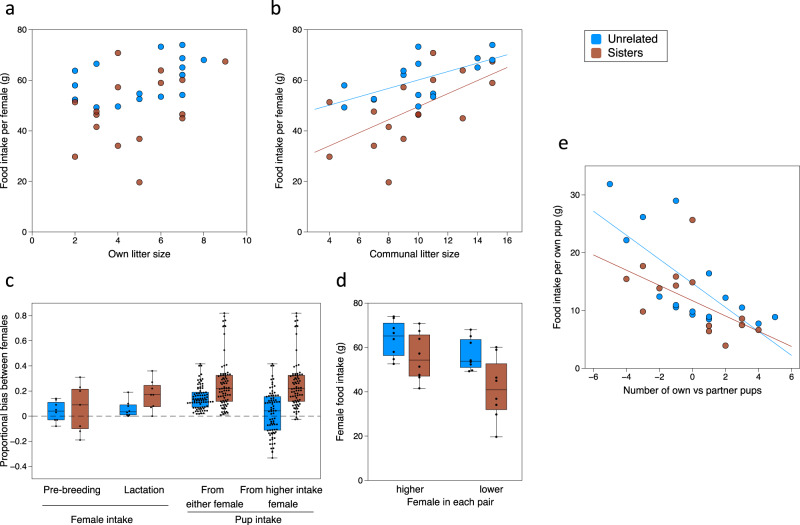
Bias in female investment and cost per own offspring reared within sister or unrelated pairs. Food intake (milk production) of individual females did not depend on own litter size (**a**) but on the combined litter size (**b**). Proportional bias in individual female food intake and in investment gained by pups from each female (regardless of maternal relatedness) was higher in sister (red) than unrelated (blue) pairs during lactation (**c**). Female energetic investment, assessed as food intake over the 7 d labelling period, was higher in unrelated females whether they had the higher or lower intake within each pair (**d**). Female energetic investment per own pup reared according to the difference in the number of own versus partner’s pups in the communal nest (**e**). Data are for individual females (circles) in *n* = 8 pairs of sisters and *n* = 8 pairs of unrelated females (**a**, **b**, **e**), or median bias ± IQR boxes with whiskers showing full range for females or individual pups (**c**, **d**). Regression lines shown where there was a significant relationship (solid lines).

Next, we address differences in individual investment between partners nursing communally and test whether sisters or unrelated females are more egalitarian. Individual females adjusted their food intake according to the total number of pups in the communal nest (*p* =  0.0007) and relatedness between the two females (*p* = 0.0011), regardless of the number or proportion of own pups in the nest (*p* = 0.26, Supplementary Table 2; Fig. 5a, b). There was also no correspondence between the proportion of pups belonging to a focal female (randomly selected in each pair) and the proportion of milk investment that pups gained from that female, regardless of whether or not females were related to partner pups (own pups: *F*_1,13_ = 0.09, *p* = 0.77; all pups: *F*_1,13_ = 0.13, *p* = 0.72). As a result, females with a smaller litter than their partner made a greater investment per own offspring raised, in agreement with findings from previous studies^19,49^. However, the bias in investment between partners differed between sisters and unrelated females.

In agreement with our prediction that sisters should be less egalitarian because a sister is more likely to compensate for an underinvesting partner, food intake to produce milk was more biased between two sisters than between unrelated partners nursing communally (*F*_1,14_ = 5.27, *p* = 0.038). This greater bias in food intake between sisters emerged only during lactation (Fig. 5c) and translated into an even stronger bias in milk gained by pups from the sister with higher food intake within each pair (Fig. 5c; bias between sisters vs unrelated, χ^2^ = 7.28, 1df, *p* = 0.007). Our analysis underestimates the bias we observed between sisters, as one pair produced a small communal litter of three pups that was fed only by the mother of one pup, so was eliminated from our dataset of communal nursing prior to analysis. However, although some sister partners made particularly low investment (Fig. 5b), the sister with highest food intake within each pair still ate only as much as a female with the lowest food intake in unrelated pairs (Fig. 5d). Thus, neither female in sister pairs had to expend more energy to raise the same weight of pups as those in unrelated pairs, even though effort among sisters was more unequal.

Neither the bias in food intake between cooperating females nor the investment received by pups was influenced by which female gave birth first (food intake: *p* = 0.58; investment: *p* = 0.81) or by the pre-breeding weight difference between females (food intake: *p* = 0.60; investment: *p* = 0.64).

### Own pups are less costly when reared with a sister partner despite skewed investment

Although sister pairs required less food to produce the same number and weight of pups as unrelated females, the greater bias in energetic investment within sister pairs combined with unequal numbers of own pups reared by each partner means that one sister paid higher costs per own pup reared in the communal nest than her partner. Unrelated pairs shared the milk investment costs more evenly but still reared unequal numbers of pups and paid higher overall energetic costs than sister pairs. This led to over an eight-fold range in the energetic costs paid per own pup reared communally by individual females, ranging from 31.9 g food intake per pup over the 7 day labelling period by an unrelated partner down to just 3.9 g intake per pup by one sister partner (Fig. 5e). The difference in number of own versus partner pups reared and the total communal litter size both substantially influenced the energetic costs per pup invested within both sister and unrelated pairs (*p* < 0.0001, Table 5). Each additional own pup above the number of partner pups reduced a female’s cost per own offspring while the partner paid higher costs. Larger total communal litter sizes also led to an apparently reduced cost per own pup over the labelling period, but this was because female investment did not increase proportionally and pups grew more slowly (see above), leading to reduced weaning weights but more pups^51^. However, relatedness between cooperating females also had a significant impact, with females paying lower costs per own pup reared with a sister despite the skew in investment within sister pairs (*p* = 0.010; Table 5). On average, females needed 15.0 ± 0.9 g of food per own pup reared communally with an unrelated partner compared to 11.4 ± 0.9 g per own pup reared communally with a sister (measured over the 7d labelling period). This represents a 31.6% average increase in energetic cost per own pup reared communally with an unrelated female compared to a sister. Some individual females paid extremely high costs per own pup when their unrelated partner had more pups in the communal nest than they had, but even those with a larger litter than their partner paid more per pup than sister partners with the larger litter (Fig. 5e). Thus, when rearing pups communally, females benefited directly from substantially lower energetic costs per own pup reared with a sister rather than with an unrelated partner, even when their investment was often exploited by the sister partner.

**Table 5:** Individual female energetic cost per own offspring raised is lower when rearing pups communally with a sister compared to an unrelated partner, whether females have more or fewer pups than their partner.

Effect^a^	Fixed effect	χ^2^	df	*p*^b^
Communal litter size	−1.10 ± 0.20	17.40	1	**<0.0001**
Difference in own minus partner pup number	−1.76 ± 0.26	28.71	1	**<0.0001**
Relatedness between females	−3.57 ± 1.28	6.55	1	**0.010**

^a^Mixed-effects model of food eaten by female over 7d labelling period per own pup raised to day 14 (g), including nest as a random effect. Data in Fig. 5e and Supplementary Data 1.

^b^Values in bolded text are statistically significant (*p* < 0.05).

## Discussion

By using a dual isotopic tracer approach to track and quantify individual milk investment during communal rearing by female house mice, our study has revealed previously undetected kinship effects arising at several levels. At the whole nest level, pairs of unrelated female cooperating partners required significantly more energy to achieve the same weight of pups compared to cooperating sisters (18% more on average), indicating greater efficiency of cooperative rearing between close relatives. Pups gained milk from both cooperating mothers in proportion to their milk production (determined by each female’s relative food intake), but pups gained slightly less provision from the partner mother than expected from her food intake. Pups younger than their communal nestmates were competitively disadvantaged in gaining access to milk from an unrelated partner mother, leading to reduced growth of younger pups in large communal litters. Considering investment by individual females, there was greater skew in the energy invested in the communal brood between two sisters than between unrelated partners, matching the prediction that females should be more willing to compensate for underinvestment by a close relative. However, as the energetic cost of communal rearing was lower for sister partners overall, the cost that individual females paid per own pup reared was lower for both cooperating sisters, whether females had more or fewer pups than their partner. On average, females paid nearly one third higher costs per own pup reared with an unrelated partner, though this could be substantially higher for a female with few pups.

Consistent with previous studies^19,20^, we found no evidence that cooperating partners attempted to adjust or bias investment according to the proportion of own offspring in the communal brood, even if there were up to four times as many unrelated pups that provided no genetic benefit to the female. The amount of milk that pups gained from each partner mother was mostly determined by the amount of food eaten by each female to produce milk. However, the small but significant bias for gaining less milk from the partner mother than predicted by her relative food intake revealed that there was some kin discrimination in pup access to milk. Pups in second-born litters under high competition were particularly disadvantaged in the amount of milk gained from an unrelated partner, achieving low weight despite higher food intake among unrelated partners. It is unlikely that this was due to active nepotism in milk delivery by mothers. Young offspring typically do not provide parentage cues that allow potential sires, or partner mothers in communal nests, to discriminate against them^57–60^. Consistent with this, house mouse mothers do not discriminate between own and other offspring of similar age in other aspects of maternal care^39,43^. Even if own offspring could be distinguished, targeting milk to individuals in a mixture of hungry offspring within communal nests would be very difficult to achieve^14^. Instead, imprinting of offspring on maternal odour is most likely to provide a mechanism of kin discrimination that is consistent with our findings. Fast attachment to teats during scramble competition among altricial mammalian pups^61,62^ blocks the availability of nipple cues to other competing pups. The ability to find teats and initiate sucking efficiently is odour-driven. Some species that do not nurse communally have evolved a generic species-specific mammary pheromone that stimulates rapid searching and efficient nipple attachment by newborns (e.g. rabbits^63^, humans^64^). By contrast, mice do not use a shared mammary pheromone, but pups imprint on own mother’s amniotic odour in utero, and on maternal salivary odour when cleaned by the mother immediately after birth^50^. Mothers spread these imprinted odours on their nipples, with suckling greatly delayed if these attractive odours are washed off^50,65^. Subsequently, experience of a milk reward during suckling reinforces an additional learned attraction to milk odour^50,65^, which is similar among females at a similar stage of lactation^52^, and will increase the efficiency of blind pups to gain milk from own and other lactating mothers in communal nests. However, maternally imprinted cues provide pups with an advantage in finding and attaching quickly to own mother’s nipples over unrelated pups that have imprinted on a different mother’s odour. Our findings suggest that a maternal imprinting strategy may have evolved as a cryptic form of kin discrimination to help pups gain their own mother’s investment under competitive conditions, even if there are bigger and more competitive offspring in the nest. This resulted in a relatively small bias in milk gained from own or partner mothers overall, as hungry pups were clearly successful in gaining milk from both mothers according to their milk production. However, the poorer growth of pups in second born litters that achieved the least success in gaining milk from a partner mother indicates that unrelated pups that were younger than competing littermates experienced increased costs that could not be compensated by increased intake of own mother milk when competition was high.

Pups in sister nests also showed a slight bias in gaining milk from their own mother, but they were generally able to access the milk they required from an aunt even under competitive conditions unless the aunt was investing little in pup feeding. Partners that are littermate sibs share very similar odours, due both to shared genes^66,67^ and shared pre- and post-natal environments^68^. Teat recognition has not yet been tested in a communal nursing scenario, but strong odour similarity means that pups imprinted on own mother’s odour are likely to also recognize teats from such close maternal relatives. Consistent with this, females strongly prefer communal nesting partners that share the most similar odours with themselves^46^, especially those that share the same inherited profile of major urinary proteins (MUPs) that shape individual-specific odour profiles^47,67^. Further, females are more likely to choose to nest communally when the degree of relatedness between nestmates is high^30^.

The main impact of kinship was that sister partners required significantly less food to rear the same weight of pups as unrelated partners, leading to substantially lower energetic costs per own offspring reared than for unrelated partners. Thus, choosing sisters as communal nursing partners provides direct benefits. We saw no evidence of more agonistic interactions or increased social dominance among unrelated partners that could have accounted for such increased energy expenditure. While we cannot completely rule out the possibility that socially tolerant but unrelated females experienced greater stress that increased their energy requirement, unrelated females ate 1.7 g more food per female per day on average than sisters while feeding pups. This amount represents a 45% increase over a female’s baseline food intake for personal maintenance prior to breeding and occurred only during lactation, making it unlikely that subtle differences in social behaviour were responsible. Instead, the big increase in energy requirement during lactation among unrelated partners was most likely a response to greater pup demand, as female mice readily respond to greater suckling by substantially elevating their food intake to increase milk production^51,55^. Among pups, milk seeking is likely to be the most energetically demanding behaviour, with efficient nipple attachment presenting a particular challenge for blind altricial offspring with limited motor skills. Improved efficiency of pup access to a closely related partner’s milk as well as own mother’s milk through imprinting on maternal odour, as discussed above, might then explain why sister partners required less food. While further work is needed to confirm energy expenditure among pups competing for milk under different scenarios, pups that are less efficient in accessing milk from an unrelated partner are very likely to use more energy trying, with both females increasing their food intake to meet this additional pup energy demand^69^. Thus, greater difficulty of gaining milk from an unrelated female among pups may substantially increase the energetic costs of communal rearing for both unrelated partners, providing a strong incentive for choosing closely related partners based on direct costs of lactation alone.

It is also possible that pups in unrelated female nests have an increased energy requirement due to less efficient thermogenesis and huddling. Pups in unrelated nests were not left alone for longer than those in sister nests and so were likely to gain similar heat input from mothers. However, while in this study we controlled for overall relatedness between pups, the average relatedness of offspring in sister communal nests would normally be higher than those of two unrelated females. This might influence the amount that pups will invest in thermogenesis when they huddle together to share heat with other pups when females are absent from the nest. In single female nests where pups may have different paternity, maternally imprinted genes in pups favour greater thermogenesis among pups that have greater relatedness through the maternal than the paternal line^70,71^. In group-hibernating alpine marmots, subordinates related to infants in the hibernaculum lost more weight, most likely through thermogenesis to keep related infants warm, while unrelated subordinates did not^72^. The impact of different degrees of relatedness on pup thermogenesis and heat sharing in communal breeding nests has yet to be explored. Pups are not expected to provide parentage cues, so they are not likely to be able to detect relatedness to other pups directly (see previous section). However, if pups detect that mothers are close relatives, they may be more willing to invest in thermogenesis as a public good compared to pups of unrelated mothers. While this would mean that pups in sister nests expend more energy in thermogenesis when huddling with nest mates than those with unrelated mothers, overall such cooperative behaviour between pups could be a more efficient energetic strategy and deserves further research.

Whatever the cause of the greater energy demand when unrelated partners reared their young communally, this substantial disadvantage is likely to be even greater under natural conditions, where foraging to meet the pups’ demand for milk will be more costly to females than in laboratory cages supplied with readily accessible food. As an increased energetic burden of rearing one litter can delay or lower performance in the next reproductive event, and can reduce female survival^73–75^, expensive cooperation with unrelated partners is also predicted to have further negative effects on subsequent reproduction and is likely to contribute to reduced lifetime reproductive success relative to cooperation between sister partners^34,42^.

Why should females rear offspring communally with unrelated partners if they risk expending more energy on rearing own and partner offspring than they would rearing own offspring alone? The main benefit of communal rearing when compared to solitary rearing in house mice appears to be joint offspring defence, as newly born altricial pups are highly vulnerable to infanticide by males and by other females^32,36,42–45^. Communal rearing might also have some additional benefits of improved thermoregulation^56^ and a reduced peak lactational load if litters are of mixed age^76^. However, for a female with a smaller litter size than her partner, communal rearing is likely to have a higher energetic cost than solitary rearing^19^. Nonetheless, if communal defence improves the likelihood that at least some of her pups will survive compared to solitary rearing, this positive impact on female fitness is likely to outweigh the potential negative effects of a higher energetic load on future reproductive fitness. When two females give birth in the same nest site, the litters are always pooled if both females have live pups^30,76^. In addition to joint cooperative defence against competitor females, this pooling of pups will provide own pups with immediate protection from the other breeding female as females are unable to discriminate between own and other pups of similar age once pooled^39,43,77^. Evidence from an 8 year study of female breeding strategies in a barn population revealed that females able to rear offspring mostly bred communally, a proportion that increased with population density (and thus pressure from competitors). Only a small proportion of females were able to rear offspring successfully on their own. Although these females reared more pups per surviving litter than those rearing pups communally, these mothers were older, larger and more experienced^40,41^. If other females did attempt to raise offspring alone, all pups were lost before researchers could detect them in fortnightly nest box checks.

Increased chance of pup survival can explain why females pool their pups but does not explain why unrelated partners invest strongly and approximately equally in their joint brood once formed, regardless of the number of own and partner pups. In the absence of indirect fitness benefits gained from helping to rear kin, or coercion that forces a subordinate partner to cooperate to their detriment, cooperative behaviour can be maintained by reciprocity or by selfish benefits gained from the behaviour^78^. Reciprocal exchanges of helpful acts among group members, which do not generate immediate selfish fitness benefits but increase the likelihood that animals will receive future benefits that compensate for the costs of initial investment, can lead to evolutionarily stable cooperation^78^. This predicts more equal investment between nonrelatives because there are no compensating indirect fitness benefits^79,80^. There is some evidence of reciprocity in allonursing non-filial offspring in herding ungulates such as reindeer^81^ and giraffe^82^. In these species, females recognise and care largely for their own single offspring but some female dyads reciprocally tolerate some allonursing of each other’s offspring, trading a similar level of investment based on allosuckling frequency and duration. Because the benefits to each partner female are correlated, both experience similar costs and gains from the exchange. However, among house mice, equal investment in a combined litter by two females can lead to substantial differences in investment per own offspring reared because individual litter sizes typically differ. In our study, the individual investment made per own offspring raised was not correlated between unrelated partners (Spearman rank correlation, *r*_s_ = 0.19, *p* = 0.65), with partners experiencing different costs and gains from the exchange. Further, in an experiment by Ferrari & Konig^83^, temporary removal of one partner for 12 h to artificially reduce one female’s lactation investment in unrelated pairs did not cause any immediate reciprocal or compensatory change in the partner female’s nursing effort. Potentially, longer-term compensation could be achieved if females with a smaller litter than their partner were likely, on average, to produce an equivalently larger litter than a partner in future communal breeding attempts. However, evidence from a large barn population, where communal rearing was by far the most prevalent breeding strategy, revealed that most females successfully reared only a single litter over their lifetime^40^. This suggests that reciprocity of investment over multiple successive litters is unlikely to stabilise communal rearing cooperation between unrelated females in this species.

Providing milk to mixed offspring in a communal nursing scenario can provide direct benefits for the female if her offspring also receive milk from other mothers, regardless of offspring number, because the female’s own offspring receive milk during lactation bouts as well as those of their partner, albeit the behaviour is more costly for a female with fewer offspring than her partner. As both unrelated partners contribute to nursing their communal offspring, this would be considered as reciprocity by some recent definitions due to the reciprocal exchange of service^78^. Reciprocal direct benefits combined with the constraint that females are unable to discriminate between offspring may explain why both unrelated partners make an equal and strong contribution to their communal offspring. Females adjust current milk investment flexibly according to the number and size of offspring by responding to suckling demand. This is likely to be the best strategy for females to maximise the growth and survival of their own offspring, whether they rear their pups solitarily or in a communal brood. Any reduction in response to hungry pup demand will reduce input into own pups in a situation where an unrelated partner is not predicted to compensate for any substantial under-investment. Indeed, we can speculate that the poorer ability of smaller pups to compete for investment from an unrelated partner found in the current study might help to drive equal and strong individual energetic investment when unrelated mothers rear their offspring communally, as reduction in individual investment could differentially harm the female’s own pups. Further research is needed to test whether direct fitness benefits are sufficient to explain the consistently strong and equal investment when familiar unrelated females pool their offspring.

Despite strong investment by both unrelated partners in their communal litter, females were not protected against exploitation by their unrelated partner: pups in the litter that was born second in a communal nest gained less investment if both unrelated partners had large litters (due to competitive disadvantage), while females with a smaller litter than their unrelated partner experienced a very poor cost to benefit ratio from communal rearing. Further, as substantially increased energetic costs among unrelated partners were not compensated by indirect genetic benefits, females will be under strong selective pressure to cooperate preferentially with a sister when possible. Communal rearing has evolved between unrelated partners in some bird species, but only where there are mechanisms that prevent or reduce individual exploitation: egg laying is synchronized between partners and any eggs laid before all group members have started to lay may be destroyed^25–27^. As birds are constrained by the ability to produce a maximum of one egg per day, this ensures that each individual contributes a similar number of age-matched eggs to the communal nest and has similar potential gain. Communal rearing in these avian species has evolved because chick survival from improved nest defence provides direct fitness benefits to all partners regardless of relatedness compared to nesting independently, while the laying strategy limits individual exploitation. By contrast, female mammals are constrained to give birth to their whole litter simultaneously, but numbers of offspring per litter in polytocous species can vary due to many factors that affect parental fecundity. There is some limited evidence that female house mice that give birth second in a communal nest might cull some offspring from the first-born litter before giving birth themselves, although selective culling does not occur once offspring are mixed and parentage cannot be distinguished^20^. This was not evident in our study. However, when we established partner pairs that reared their offspring communally for this study, some females lost their entire litter in the first few days after birth (11%) so were not included in the study. This level of newborn litter loss is not unusual among singly-housed females breeding in our wild-stock mouse colony, but we noted that full litter loss occurred in some familiar unrelated pairs but not in familiar sister pairs in this study (Fisher’s exact test, *p* = 0.06). Some females might have decided to avoid communal nursing when housed with a non-relative despite social tolerance of the partner, with the newborn offspring disappearing, presumed eaten. We could not tell whether litter loss was due to the pups’ own mother, to her partner or to both females as mice construct fully enclosed nests. Given the higher costs of communal rearing with a non-relative, females might be able to benefit from regaining the smaller amount of energy invested in newborn pups and delay their reproduction until they can rear pups alone or with a close relative.

When cooperating with sisters, the substantial bias in investment between some sisters was consistent with the prediction that partners are more likely to compensate for underinvestment (or even abandonment) by a close relative, due to indirect genetic benefits from helping to rear related offspring as long as the cost is not too high^17^. Indeed, in one additional pair of sisters in our study, only one female fed a very small joint litter without any detectable lactational investment from her sister, which might be a case of conspecific brood parasitism. From a theoretical perspective, this tactic can be beneficial for both host and parasite when mothers are related and parasitism costs are not high (in this case, sisters had a very small joint litter), and is a kin-related parasitic tactic that has been reported in a range of bird and insect species^17^. When offspring are easily able to access milk from own or related mothers, differences in quality or condition between females could result in unequal effort or pup demand. This can increase the energetic costs of communal rearing for some individuals substantially above that required to provision only their own pups, even though sisters paid lower costs than unrelated females. In house mice, energetic costs of communal rearing for individual females are higher on average than for solitary rearing^19^, but pups in communal nests grow faster and females gain additional benefits such as improved offspring defence that might increase the likelihood of pup survival^43^, which is likely to outweigh some increase in energetic cost. Studies of a stable free-range population of house mice have shown strong bias for communal rearing when local population density is high, particularly among females with low body mass, suggesting that this is a reproductive tactic that allows at least some reproductive success under suboptimal conditions^41^. However, choice of communal or solitary rearing is condition-dependent: while 69% of all litters were reared communally with familiar relatives, the choice to rear offspring cooperatively decreased with increasing female age and weight^30,41^. Older females typically produce fewer offspring per litter, while heavier females produce more milk^19,51,84^. As age and weight differences between females in free-range populations are much greater than between the age-matched female partners used in our study, older heavier females may be at greater risk of exploitation from smaller, weaker or less experienced partners, in communal rearing systems where reproductive success of breeders is not controlled through social dominance. To avoid possible exploitation, females in communal rearing systems may evolve mechanisms to select partners that are closely matched as well as closely related^30,48^, though this has received little attention to date.

Our study highlights a key difference between reproductive cooperation through mutualistic communal rearing or through cooperative breeding with non-breeding helpers. In helper breeding systems, helpers boost the reproductive success of breeders by provisioning their offspring^85^ and, in doing so, reduce the costs of offspring provision and care for the dominant breeders by lightening the breeder’s load^86,87^. Subordinates may gain some reproductive success in such systems; however, dominant breeders generally control group membership, resource access and offspring survival such that subordinate breeding does not impose a major cost on dominant reproductive success and creates high reproductive skew in favour of dominant breeders^88^. By contrast, mutualistic communal rearing systems have much lower reproductive skew but bring a risk of substantially increased costs for some breeding partners: females with fewer or younger (less competitive) offspring, or those in better condition, risk paying greater costs when breeding cooperatively, even with close relatives. However, group living and cooperative breeding are obligatory for survival in some species, such as banded mongooses (*Mungos mungo*), which have evolved a hybrid helper and communal rearing system in which multiple females breed in tight synchrony^89^. When reproductive competition becomes too high, a cohort of older dominant females forcibly evicts younger subordinates^90^, but evictions among breeding age subordinates are targeted to those most closely related to themselves^91^. A suggested explanation for this negative kin discrimination is that closely related subordinates might be easier to evict^91^. However, as in house mice, pups appear to be suckled indiscriminately by breeders^92^. If mongooses exhibit covert kin discrimination during lactation similar to that demonstrated in our study, offspring of closely related subordinates may pose a greater threat because they are better able to access milk from larger related dominant females under competitive conditions. Such competition could strongly impact the reproductive success of dominant animals, as pups that are heavier on emergence from the den have greater survival to independence^93^. Further work is needed to understand the balance between cooperation, competition and relatedness in communal breeders, and the mechanisms that underpin this, particularly when breeders can gain inclusive fitness benefits from helping relatives but potentially could gain more from targeting investment to own offspring.

In conclusion, by developing a method to accurately track individual maternal investment, our study has revealed important kin discrimination during communal rearing that has not been possible to detect previously. This is likely to provide a strong driver for selecting close kin as cooperative partners where possible. At least some of this discrimination may arise from an imprinting mechanism that helps offspring gain investment from own mother and closely related partners, particularly when competing with offspring from unrelated mothers. Significantly greater energetic efficiency of pup rearing between close relatives is a direct benefit of cooperating with kin, with both related partners experiencing substantially reduced costs for each own offspring raised compared to communal rearing by unrelated breeding partners, in addition to any indirect genetic benefits gained from helping to rear closely related offspring. Our study suggests that strong preference for communal rearing with relatives is not simply a consequence of kin structuring in species that have evolved mechanisms that favour investment in own and closely related offspring; instead, the benefits of choosing close kin as rearing partners may provide a strong driver to maintain kinship grouping between adult females where communal breeding is advantageous or cannot be avoided. Nonetheless, mutualistic communal rearing systems still bring a risk of increased energetic costs for some breeding partners if investment is not proportional to the number or weight of own offspring reared. The extent to which animals attempt to exploit the investment of related breeding partners, or evolve mechanisms that minimise such exploitation to maintain a stable system of mutual cooperation, remains to be determined.

## Methods

All animal care protocols were in accordance with the University of Liverpool Animal Welfare Committee requirements, with EU Directive 2010/63/EU and UK Home Office guidelines for animal care. Tissue samples from live animals were obtained under UK Home Office licence under the Animals in Scientific Procedures Act 1986, according to best practice guidelines. The University of Liverpool Animal Welfare Committee approved the work.

### Subjects

Subjects were captive-bred female and male *Mus musculus domesticus* derived from ancestors captured from six populations in the northwest of England UK. From weaning, females were housed in 45 × 28 × 13 cm cages (MB1, North Kent Plastics, UK) in small single-sex family groups of 2-4. Males (used as sires) were singly housed in 43 × 11.5 × 12 cm cages (M3, North Kent Plastics, UK) as wild-stock males become highly aggressive towards other males once adult. Mice were maintained on Corn Cob Absorb 10/14 substrate with paper wool nest material and *ad libitum* access to water and food (5002 Diet, LabDiet, St Louis, USA). Cardboard or plastic tubes, plastic hanging baskets (Datesand Ltd, Manchester, UK), red plastic mouse houses (Tecniplast UK Ltd) and/or cardboard boxes were provided for home cage enrichment. Mice were also regularly exposed to scents from other cages of mice for olfactory enrichment. Animals were housed on a reversed 12:12 h light cycle with lights off at 08:00, temperature 20–22 °C and relative humidity 45–65%. All females were individually marked with an RFID tag inserted under the skin at the nape of the neck.

We aimed to compare communal nursing in eight pairs of littermate sisters and eight pairs of familiar unrelated females that were matched for age and prior breeding experience, and where litters within each pair were born within 5 days (82% of females that chose to nest communally in a free-living barn population joined another litter that was up to 5 days old^30^). To achieve this required the establishment of 23 sister and 26 unrelated pairs (98 females), as some females did not breed within the required time period (26/98, 27%), two females were withdrawn from the experiment due to ill health (2%), and not all litters born survived (8/70, 11%). In addition, a technical feeder fault led to cross contamination of labeled diets in two unrelated pairs that could not be used, and one sister communal litter did not involve communal nursing as pups were fed by only one sister so were excluded.

Littermate sisters were used to ensure that females in related pairs had the full range of genetic and environmental cues (being reared together) that house mice can use to discriminate close relatives, while unrelated females shared neither genetic nor environmental cues corresponding to potential relatedness. Littermate sisters were separated from other family members, and unrelated pairs co-housed in MB1 cages, for at least one month prior to breeding. Not all sister pairs of wild-stock house mice are socially compatible once adult, while adult females may tolerate some unrelated females encountered in adulthood but not others. Only pairs of females that were socially tolerant of each other were used (no aggression observed after initial introduction). Across all pairs of females that were mated to males to produce communal litters for this study, a similar proportion of females gave birth in sister pairs (33/46, 71.7%) and unrelated pairs (41/52, 78.8%). Both females in a pair produced a litter in a similar 57% (13/23) of sister and 62% (16/26) of unrelated pairs, while only one female gave birth in 30% of sister and 35% of unrelated pairs. Among pairs where both females gave birth within the required time period (5 days), both litters survived in fewer unrelated pairs (10/16) than in sister pairs (9/9), although this difference did not quite reach statistical significance (Fisher’s exact test, *p* = 0.057). First-born litters disappeared before the second female gave birth in 4/16 unrelated pairs, and neither litter survived in 2/16 unrelated pairs. As female mice will often kill and ingest their own newborn offspring within the first few days after birth, it was not known whether offspring were killed by the mother, partner or both as disturbance during this sensitive time was kept to a minimum by only checking nests for litters once per day.

Where necessary, replacement pairs were selected from our breeding colony to ensure that data for each pair of sisters was matched by data from an equivalent unrelated pair of the same age (within one month) and prior breeding experience. Overall, a similar proportion of all females in sister pairs (13/46, 28.3%) and unrelated pairs (19/52, 36.5%) failed to rear offspring due to not producing a litter or losing the litter within the first few days (χ^2^ = 0.76, *p* = 0.38). In the final dataset of 16 pairs that reared offspring communally so were used in this study, sister and unrelated pairs did not differ in their mean weight (mean ± sem: 21.3 ± 0.6 g, *F*_1,14_ = 1.57, *p* = 0.23) or weight difference within pair prior to breeding (3.1 ± 0.5 g, *F*_1,14_ = 0.62, *p* = 0.44), the age difference between litters within the same communal nest (2.4 ± 0.4 d, *F*_1,14_ = 0.10, *p* = 0.75), or the mean age of pups at day 14 (12.8 ± 0.2 d, *F*_1,14_ = 0.28, *p* = 0.60). Thus, both females and communal nests were well matched between unrelated and sister pairs.

### Feeder training

Prior to breeding, the two females within each pair were trained to use separate feeders in an adapted MB1 cage, using unlabelled diet. Two automated feeders custom-built for the project (Francis Scientific Instruments, Cambridge, UK) were attached to opposite sides of the cage through external tunnels (42 × 32 mm), with an acrylic nestbox suitable to accommodate two females and their combined pups (116 × 116 × 89 mm, lid perforated by multiple holes for good ventilation) attached to the short edge of the cage midway between the feeders (Fig. 2). Feeder access was controlled by RFID tag readers around each access tunnel that were programmed to recognise different females. When the incorrect RFID was detected, a servomotor moved the hinged food hopper away from the feeder grille at the end of the tunnel such that females could only gain food from their own feeder. Note that the default position always allowed food access so that any technical problems with the system would not deprive females of food. To train females to feed reliably from their own feeder, pairs were housed in the adapted feeder cage for two days with feeders in the default accessible position and additional food placed in a shared cage hopper. Feeders were then switched on with supplementary food still available in the shared cage hopper for a further two days, before all supplementary food was removed on day 5. Females and food were then weighed daily for 11–14 days to ensure they were eating reliably. If females were initially reluctant to use the feeders, supplementary peanuts were added until they were taking food reliably. If any females were not used immediately for communal nursing, they were given refresher experience of the feeders for 7–8 days prior to use.

### Communal nursing experiment

Females were housed separately with unrelated males for seven days to mate. Pairing with males was staggered by two days between the two females in each pair to promote a small age difference of up to 5 days between their litters. Females were pre-exposed to their mate through a mesh partition in an MB1 cage for 3 days prior to mixing to stimulate oestrus and attraction to the male^94^, so that females would mate within the required time period. To control the degree of relatedness between litters in communal nests while the relatedness between females (and between females and pups) was manipulated, sisters were mated with males that were unrelated to each other while each unrelated pair was mated to two brothers (unrelated to the females). Thus, all communal litters consisted of full sibs and maternal or paternal cousins. It should be noted that this design could not control for any differences in competition between litters due to maternal versus paternal relatedness, which can influence kin discrimination in some species. For example, while tadpoles prefer both maternal and paternal half-siblings over non-siblings, they prefer maternal over paternal half-siblings^95^. Larvae of the solitary parasitoid *Aleochara bilineata* avoid superparasitism of hosts that are already infected with a full sib larva, where only one larva can survive. They similarly avoid competing with cousins related through their father but not with those related through their mother; this is most likely due to a maternal imprinting mechanism that switches off female-transmitted genes responsible for detection of kinship signals, although the benefit of not recognising maternal cousins in this situation is uncertain^96^. However, in species such as house mice, where young offspring are vulnerable to potential infanticide by unrelated adult males and females, young are not expected to express either maternal or paternal parentage cues that could be used in kin discrimination^57–60^.

Females reunited after mating were housed in an MB1 cage fitted with two individual feeders and an external plastic nestbox containing shredded paper (see feeder training). A pair of automated RFID readers (Francis Scientific Instruments, Cambridge, UK) around the tunnel leading to the nestbox logged the time, date and RFID code each time a female passed through^47^. This allowed us to monitor the time spent in the nest by each female alone or together, calculated using a custom syntax written in SPSS (available on request). Females were fed on Certified Rodent Diet 5002 separately labelled with 50% [2H4] or [2H9] lysine dihydrochloride (referred to as d4 and d9 diets) for 7 d once both females had given birth and first born litters were 7 d old. To make 400 g batches of labelled diets, 7.05 g of [2H4] or [2H9] lysine was dissolved in 400 ml RO water, stirred in to 400 g unlabeled 5002 diet containing 1.18% lysine and left for 2 h. A further 62.5 ml RO water was then added and stirred aggressively, left for a further 1 h, added to a blender using 62.5 ml RO water to rinse the beaker and blended for a minimum 10 minutes until the mixture resembled a thick paste. The food was transferred to a piping bag, piped onto baking paper, scored into pellets and placed in a dehydrator at 40 °C for approximately 48 h.

The amount of food remaining in each feeder was weighed daily and replenished. All pups were culled humanely when first born litters reached 14 d old, before pups started to take solid food. Pups were weighed and frozen at −20 °C before heart and other organs were dissected out for relative investment analysis. A urine sample was also obtained from each female immediately at the end of the experiment, by temporarily confining each female on a mesh grill over a clean cage for 30–60 min, to check that contamination of dietary labels was minimal.

To confirm maternal parentage, pups, mothers and sires were each genotyped at microsatellite markers in the MUP (D4NDS6, D4Mit139, D4Mit241, D4Mit164, D4Mit217, D4Mit17) and MHC (D17Mit22, D17Mit13, D17Mit234, D17Mit126) regions. Markers were chosen from those already shown to exhibit high polymorphism in wild UK house mice from the same colony^47^. DNA was extracted from pup tissue after culling, or from a 5 mm ear punch from adult mice, using a QIAGEN DNeasy Blood & Tissue Kit (QIAGEN, West Sussex, UK). Genotyping was carried out using the same protocol reported in ref. ^47^.

### Preliminary labelling experiment

We conducted an initial experiment using laboratory mice to determine the kinetics of labelling proteins in milk, pup tissues and urine, and establish the most suitable analytical strategy to accurately measure the relative milk investment received by pups. For this, we used three pairs of inbred BALB/c females, each mated to a single male to produce three communal litters (in-house bred from BALB/cOlaHsd stock originally obtained from Harlan UK). As domesticated laboratory mice are more tolerant of disturbance than wild house mice, this allowed us to sample litters on a daily basis without the risk of infanticide. Females were housed in standard MB1 cages on Corn Cob Absorb 10/14 substrate with paper wool nest material and red plastic mouse houses (Tecniplast UK Ltd) for breeding, as well as hanging baskets for enrichment; water and Certified Rodent Diet 5002 (labelled or unlabelled) were available ad libitum. All females were fed on the same diet labelled with 50% [13C6]lysine (using the same protocol for incorporation of label given above) as we only needed to track the labelling of pups in this pilot experiment, regardless of which mother provided the milk. Three communal litters were used to allow sampling of two pups per day while avoiding excessive depletion of litters that might influence the dynamics of labelling. Females were switched on to labelled diet four days after pups were born and remained on the labelled diet for 6 d. During the labelling period urine samples were obtained from at least two of the mothers each day. Two pups were also humanely culled each day to sample urine, milk from the stomach, and other pup tissues, frozen at −20 °C until protein analysis.

### Mass spectrometry analysis

Pup tissue, maternal milk and urine samples were digested with trypsin or endopeptidase LysC^97^. Samples were analysed using an Ultimate 3000 RSLC™ nano system (Thermo Scientific, Hemel Hempstead) coupled to a QExactive-Hf™ or a QExactive mass spectrometer (Thermo Scientific). The sample was loaded onto the trapping column (Thermo Scientific, PepMap100, C18, 300 μm × 5 mm), using partial loop injection, for seven minutes at a flow rate of 4 μL/min with 0.1% (v/v) formic acid. The sample was resolved on the analytical column (Easy-Spray C18 75 µm x 500 mm 2 µm column) using a gradient of 97% A (0.1% (v/v) formic acid) 3% B (99.9% (v/v) acetonitrile, 0.1% (v/v) formic acid) to 60% A, 40% B over 60 minutes at a flow rate of 300 nL min^-1^. The data-dependent program used for data acquisition consisted of a 70,000 resolution full-scan MS scan (automatic gain control (AGC) set to 10^6 ^ions with a maximum fill time of 200 ms). The three most abundant peaks were selected for MS/MS using a 35,000 resolution scan (AGC set to 10^5 ^ions with a maximum fill time of 50 ms) with an ion selection window of 1.2 m/z and a normalised collision energy of 30. To avoid repeated selection of peptides for MS/MS, the program used a 15 s dynamic exclusion window. Peak areas under the curve for both the light and heavy peptide forms were extracted using Skyline (version 4.1.0.11796)^98^ in order to calculate isotopic enrichment (see Supplementary Note 1).

### Calculation of relative investment gained by pups

To assess the relative contribution each mother made to the pups during the labelling period, pup heart proteins were recovered, digested with trypsin and analysed by LC-MS/MS. From the pilot, [^13^C]lysine experiment, we selected a protein with an intermediate rate of turnover/isotope accretion that would be insensitive to hour to hour variation in isotope input but at the same time, would accumulate sufficient label for accurate quantification of relative abundance. Further, we used a protein/peptide that was observed in every sample, to obtain a coherent data set. The analysis was based on a peptide NGDTITIK from fatty acid binding protein, which yielded high quality extracted ion chromatograms and spectra ions at M ([^1^H]lysine), M + 4 Da ([^2^H_4_]lysine) and M + 9 Da ([^2^H_9_]lysine). Because each of these three peptides also had the natural 13 C isotopomer envelopes, it was necessary to correct for the small element of ‘isotopomer spillover’ from one peptide profile to another. This is explained in detail in Supplementary Note 1. The isotopomer distribution was calculated as in Supplementary Fig. 7 using an on-line tool. From this peptide specific profile, the ‘isotopomer spillover’ could be calculated (Supplementary Fig. 8).

Having made the isotopomer correction, the relative investment from the two mothers was assessed by measurement of the [^2^H_4_]lysine (‘d4’) and [^2^H_9_]lysine (‘d9’) peak areas. For this calculation, we needed to know the extent to which mothers had acquired slight access to the other diet and correct for this. This calculation used a simple spreadsheet tool (Supplementary Software 1, Supplementary Fig. 9).

### Statistics and reproducibility

Analyses were performed in SPSS (IBM version 27) or R (v. 3.6.2)^99^. All statistical tests are two-tailed. Measures are taken from distinct individual pups, individual mothers or pairs of mothers as indicated below. Summary data are given as mean ± *SEM* unless otherwise indicated. For all analyses detailed below, we examined the distribution of residuals to check for approximation to normality and confirmed the good fit of data to each model using Shapiro-Wilks tests (Supplementary Figs. 10–17 contained in Supplementary Note 2). Non-significant interaction terms were dropped from models.

The overall success of communal nests (data per nest) was compared between sisters and unrelated pairs using ANOVA (number of pups born or surviving to 14 days after first pups born; age gap between first and second-born litters, mean age of pups at day 14). Analysis of covariance (ANCOVAR) examined the effects of relatedness between partners, communal litter size, age of second born litter and mean weight of partners prior to breeding on the weight of pups achieved in each communal litter (total and mean weight per pup, Supplementary Table 1), and on the total food intake by each pair of mothers over the 7 d labelling period (Table 1). ANCOVAR also examined whether relatedness between partners influenced the relationship between food intake and total weight of pups by day 14, taking age of second born litter and mean weight of female into account (Table 1). ANOVAs assessed the effect of relatedness on the change in daily food intake per pair during lactation (repeated measures over 7 days of labelling), and on food intake prior to breeding once females had been trained to use feeders.

Factors influencing the proportion of milk gained by each pup from own or partner mother were analysed using linear mixed effects models, including the identity of each communal nest and mother as random effects (lmer function in lme4 package in R^100^). To assess the significance of each fixed factor, or interaction between fixed factors, likelihood ratio tests compared models with or without the specific factor or interaction using the anova function. To assess whether pups gained more milk from own than from the partner mother, we compared the proportion of milk gained from a focal female in each pair (assigned randomly by coin toss) according to whether this was own mother or partner. As food intake determines the amount of milk a female can produce, the proportion of total food eaten by each pair during labelling that was due to the focal female was included as a fixed factor in the model along with relatedness between the partners (Table 2). To look at the effect of competition between pups on the proportion of investment gained from the partner mother (Table 3), whether pups were from the first litter (older pups) or second litter (younger pups) born in the communal nest and communal litter size were included as fixed factors, along with the proportion of total food eaten by the partner mother and relatedness of the partner mother to the pups. Where both litters were born on the same day (one sister pair, one unrelated pair), both were assigned as first born since they did not differ in age and development. As there was a significant interaction between all four factors in this model, separate models were run post-hoc to examine the effects of communal litter size, partner relatedness and food intake on first and second born litters separately, and on related and unrelated second born litters separately. To correct for multiple testing across four sub-models, significance was assessed against a Bonferroni corrected *p* value of 0.0125 (Table 3).

To examine whether competition and the proportion of milk gained from different mothers influenced the body weight achieved by pups by day 14 (Table 4), separate models were run for first and second-born litter pups as all first litter pups were the same age (14 days) while age of second litter pups varied (9–13 days old) and age is a major factor influencing body weight among growing pups. The models included all variables likely to influence pup body weight as fixed factors (pup age at day 14 for second-born litters only, communal litter size, total female food intake, proportion of milk gained from partner female minus proportion of the pair’s total food intake eaten by the partner, own mother body weight, partner mother body weight, pup sex and relatedness of partner mothers), with mother included as a random effect. The distribution of residuals for first born litter pups revealed a strong outlier (one male pup from unrelated nest B, ID 36337, weight 10.6 g) that was much heavier than other pups in the same litter (7.16 ± 0.14 g) and could be an erroneous data point (Supplementary Fig. 13a, b in Supplementary Note 2). This pup was removed from the model shown in Table 4 to meet assumptions of normality (Supplementary Fig. 13c, d), but removing this male from the model made no difference to conclusions.

To look at investment by individual partner females, a linear mixed effects model examined whether a female’s food intake over the 7 d labelling period depended on the communal litter size or number of own pups in the nest (surviving to day 14), with relatedness between partners included as a fixed effect and nest as a random effect (Supplementary Table 2). The relationship between the proportion of pups belonging to a focal female in each nest (randomly selected) and proportion of milk received from that female was explored using ANCOVAR. The effect of relatedness on the absolute bias in food intake between partner females was examined using ANOVA, and on the bias in pup intake from partner females using a linear mixed effects model to take mother and nest into account as random effects. The effect of birth order or pre-breeding weight difference between females on their bias in food intake or investment received by pups from a randomly selected focal female was also examined using ANCOVAR, with relatedness and communal litter size included as covariates. Finally, we divided the amount of food that each female ate over the 7d labelling period by the number of own pups reared to assess the energetic cost paid per own pup. A linear mixed effects model examined the effect of relatedness between females whilst taking into account communal litter size and the difference in number of own pups minus the number of partner pups in the nest, with nest identity included as a random effect (Table 5).

### Reporting summary

Further information on research design is available in the Nature Portfolio Reporting Summary linked to this article.

## Supplementary information

Supplementary Information

Description of Additional Supplementary Files

Supplementary Software 1

Supplementary Data 1

Reporting Summary

## Data Availability

The mass spectrometry proteomics data for the main study (relative investment) have been deposited to the ProteomeXchange Consortium via the open access PRIDE partner repository^101^ with the dataset identifier  https://www.ebi.ac.uk/pride/archive/projects/PXD019578. In addition, the milk pilot study proteomics data have been deposited at the same site with identifier https://www.ebi.ac.uk/pride/archive/projects/PXD019586. All other data needed to evaluate the conclusions in the paper are provided in Supplementary Data 1.

## References

[1] Solomon, N. G. & French, J. A. *Cooperative Breeding in Mammals*. (Cambridge University Press, 1997).

[2] Koenig, W. D. & Dickinson, J. L. *Cooperative Breeding in Vertebrates*. (Cambridge University Press, 2016).

[3] Rubenstein, D. R. & Abbot, P. *Comparative Social Evolution*. (Cambridge University Press, 2017).

[4] Clutton-Brock TH (2009). Cooperation between non-kin in animal societies. Nature.

[5] Downing PA, Griffin AS, Cornwallis CK (2020). Group formation and the evolutionary pathway to complex sociality in birds. Nat. Ecol. Evol..

[6] Hatchwell BJ (2009). The evolution of cooperative breeding in birds: kinship, dispersal and life history. Philos. Trans. R. Soc. Lond. B Biol. Sci..

[7] Hamilton WD (1964). The genetical evolution of social behaviour. I. J. Theor. Biol..

[8] Cornwallis CK (2017). Cooperation facilitates the colonization of harsh environments. Nat. Ecol. Evol..

[9] Griesser M, Drobniak SM, Nakagawa S, Botero CA (2017). Family living sets the stage for cooperative breeding and ecological resilience in birds. PLoS Biol..

[10] Lin YH, Chan SF, Rubenstein DR, Liu M, Shen SF (2019). Resolving the Paradox of Environmental Quality and Sociality: The Ecological Causes and Consequences of Cooperative Breeding in Two Lineages of Birds. Am. Nat..

[11] Garcia-Ruiz I, Quinones A, Taborsky M (2022). The evolution of cooperative breeding by direct and indirect fitness effects. Sci. Adv..

[12] Taborsky M, Frommen JG, Riehl C (2016). The evolution of cooperation based on direct fitness benefits. Philos. Trans. R. Soc. Lond. B Biol. Sci..

[13] Lewis, S. E. & Pusey, A. E. in *Cooperative Breeding in Mammals* (eds N. G. Solomon & J. A French) Ch. 12, 335-363 (Cambridge University Press, 1997).

[14] Hayes LD (2000). To nest communally or not to nest communally: a review of rodent communal nesting and nursing. Anim. Behav..

[15] Vehrencamp, S. L. & Quinn, J. S. in *Ecology and Evolution of Cooperative Breeding in Birds* (eds W. D. Koenig & J. L. Dickinson) Ch. 11, 177-196 (Cambridge University Press, 2004).

[16] Wcislo, W. T. & Tierney, S. M. in *Organisation of Insect Societies* (eds J. Gadau & J. Fewell) (Harvard University Press, 2009).

[17] Andersson M, Ahlund M, Waldeck P (2019). Brood parasitism, relatedness and sociality: a kinship role in female reproductive tactics. Biol. Rev..

[18] Gerlach G, Bartmann S (2002). Reproductive skew, costs, and benefits of cooperative breeding in female wood mice (*Apodemus sylvaticus*). Behav. Ecol..

[19] Ferrari M, Lindholm AK, Konig B (2015). The risk of exploitation during communal nursing in house mice, *Mus musculus domesticus*. Anim. Behav..

[20] Ferrari M, Lindholm AK, Konig B (2016). A reduced propensity to cooperate under enhanced exploitation risk in a social mammal. Proc. R. Soc. Ser. B.

[21] Mennella JA, Blumberg MS, McClintock MK, Moltz H (1990). Inter-litter competition and communal nursing among Norway rats: advantages of birth synchrony. Behav. Ecol. Sociobiol..

[22] Federico V, Allaine D, Gaillard JM, Cohas A (2020). Evolutionary Pathways to Communal and Cooperative Breeding in Carnivores. Am. Nat..

[23] Lubin Y, Bilde T (2007). The evolution of sociality in spiders. Adv. Study Behav..

[24] Schwarz MP, Richards MH, Danforth BN (2007). Changing paradigms in insect social evolution: insights from halictine and allodapine bees. Annu Rev. Entomol..

[25] Riehl C (2011). Living with strangers: direct benefits favour non-kin cooperation in a communally nesting bird. Proc. Biol. Sci..

[26] Macedo, R. H. in *Cooperative Breeding in Vertebrates: Studies of Ecology, Evolution and Behavior* (eds W. D. Koenig & J. L. Dickinson) Ch. 15, 257-271 (Cambridge University Press, 2016).

[27] Shen, S. F., Yan, H. W. & Liu, M. in *Cooperative Breeding in Vertebrates: Studies of Ecology, Evolution and Behavior* (eds W. D. Koenig & J. L. Dickinson) Ch. 14, 237-256 (Cambridge University Press, 2016).

[28] Abbot, P. & Chapman, T. in *Comparative Social Evolution* (eds D. R. Rubenstein & P. Abbot) 154-187 (Cambridge University Press, 2017).

[29] Cornwallis CK, West SA, Griffin AS (2009). Routes to indirect fitness in cooperatively breeding vertebrates: kin discrimination and limited dispersal. J. Evol. Biol..

[30] Harrison N (2018). Female nursing partner choice in a population of wild house mice (*Mus musculus domesticus*). Front. Zool..

[31] Packer C, Lewis S, Pusey A (1992). A comparative analysis of non-offspring nursing. Anim. Behav..

[32] Rusu AS, Krackow S (2004). Kin-preferential cooperation, dominance-dependent reproductive skew, and competition for mates in communaly nesting female house mice. Behav. Ecol. Sociobiol..

[33] Mathot KJ, Giraldeau LA (2010). Within-group relatedness can lead to higher levels of exploitation: a model and empirical test. Behav. Ecol..

[34] Konig B (1993). Maternal investment of communally nursing female house mice (*Mus musculus domesticus*). Behav. Process..

[35] Konig B (1994). Fitness effects of communal rearing in house mice: the role of relatedness versus familiarity. Anim. Behav..

[36] Dobson FS, Jacquote C, Baudoin C (2000). An experimental test of kin association in the house mouse. Can. J. Zool..

[37] Mendl M, Paul ES (1989). Observation of nursing and sucking behaviour as an indicator of milk transfer and parental investment. Anim. Behav..

[38] Cameron EZ (1998). Is suckling behaviour a useful predictor of milk intake? A review. Anim. Behav..

[39] Konig, B. & Lindholm, A. K. in *Evolution of the House Mouse* (eds M. Macholan, S. J. E. Baird, P. Munclinger, & J. Pialek) 114-134 (Cambridge University Press, 2012).

[40] Ferrari M, Lindholm AK, Konig B (2019). Fitness consequences of female alternative reproductive tactics in house mice (*Mus musculus domesticus*). Am. Nat..

[41] Ferrari M, Lindholm AK, Ozgul A, Oli MK, Konig B (2022). Cooperation by necessity: condition- and density-dependent reproductive tactics of female house mice. Commun. Biol..

[42] Konig B (1994). Components of lifetime reproductive success in communally and solitary nursing house mice - a laboratory study. Behav. Ecol. Sociobiol..

[43] Manning CJ, Dewsbury DA, Wakeland EK, Potts WK (1995). Communal nesting and communal nursing in house mice, *Mus musculus domesticus*. Anim. Behav..

[44] Palanza P, Della Seta D, Ferrari PF, Parmigiani S (2005). Female competition in wild house mice depends upon timing of female/male settlement and kinship between females. Anim. Behav..

[45] Schmidt J (2015). Reproductive asynchrony and infanticide in house mice breeding communally. Anim. Behav..

[46] Manning CJ, Wakeland EK, Potts WK (1992). Communal nesting patterns in mice implicate MHC genes in kin recognition. Nature.

[47] Green JP (2015). The genetic basis of kin recognition in a cooperatively breeding mammal. Curr. Biol..

[48] Weidt A, Hofmann SE, Konig B (2008). Not only mate choice matters: fitness consequences of social partner choice in female house mice. Anim. Behav..

[49] Auclair Y, Konig B, Ferrari M, Perony N, Lindholm AK (2014). Nest attendance of lactating females in a wild house mouse population: benefits associated with communal nesting. Anim. Behav..

[50] Logan DW (2012). Learned recognition of maternal signature odors mediates the first suckling episode in mice. Curr. Biol..

[51] Konig B, Riester J, Markl H (1988). Maternal care in house mice (*Mus musculus*): II. The energy cost of lactation as a function of litter size. J. Zool., Lond..

[52] Al Ain S, Goudet C, Schaal B, Patris B (2015). Newborns prefer the odor of milk and nipples from females matched in lactation age: Comparison of two mouse strains. Physiol. Behav..

[53] Claydon AJ, Thom MD, Hurst JL, Beynon RJ (2012). Protein turnover: measurement of proteome dynamics by whole animal metabolic labelling with stable isotope labelled amino acids. Proteomics.

[54] Hammond, D. E. et al. Harmonizing labeling and analytical strategies to obtain protein turnover rates in intact adult animals. *Mol. Cell. Proteomics* **21**10.1101/2021.12.13.472439 (2022). 10.1016/j.mcpro.2022.100252 PMC9249856 35636728

[55] Fuchs S (1982). Optimality of parental investment: the influence of nursing on reproductive success of mother and female young house mice. Behav. Ecol. Sociobiol..

[56] Hayes LD, Solomon NG (2006). Mechanisms of maternal investment by communal prairie voles, *Microtus ochrogaster*. Anim. Behav..

[57] Holmes WG, Sherman PW (1982). The ontogeny of kin recognition in two species of ground squirrels. Anim. Behav..

[58] Kazem AJN, Barth Y, Pfefferle D, Kulik L, Widdig A (2018). Parent-offspring facial resemblance increases with age in rhesus macaques. Proc. Biol. Sci..

[59] Marshall HH (2021). A veil of ignorance can promote fairness in a mammal society. Nat. Commun..

[60] Richardson J, Smiseth PT (2020). Maternity uncertainty in cobreeding beetles: females lay more and larger eggs and provide less care. Behav. Ecol..

[61] Gilbert AN (1986). Mammary number and litter size in Rodentia: The “one-half rule”. Proc. Natl Acad. Sci. USA.

[62] Hudson R, Trillmich F (2008). Sibling competition and cooperation in mammals: challenges, development and prospects. Behavioural Ecol. Sociobiol..

[63] Schaal B (2003). Chemical and behavioural characterization of the rabbit mammary pheromone. Nature.

[64] Doucet S, Soussignan R, Sagot P, Schaal B (2009). The section of areolar (Montgomery’s) glands from lactating women elicits selective, unconditional responses in neonates. PLOS One.

[65] Al Ain S, Belin L, Schaal B, Patris B (2013). How does a newly born mouse get to the nipple? Odor substrates eliciting first nipple grasping and sucking responses. Dev. Psychobiol..

[66] Todrank J, Heth G (2003). Odor-genes covariance and genetic relatedness assessments: rethinking odor-based “recognition” mechanisms in rodents. Adv. Study Behav..

[67] Roberts SA (2018). Individual odour signatures that mice learn are shaped by involatile major urinary proteins (MUPs). BMC Biol..

[68] Nakamura K, Kikusui T, Takeuchi Y, Mori Y (2008). Influences of pre- and postnatal early life environments on the inhibitory properties of familiar urine odors in male mouse aggression. Chem. Senses.

[69] Fleming AS (1976). Control of food intake in the lactating rat: role of suckling and hormones. Physiol. Behav..

[70] Haig D (2008). Huddling: brown fat, genomic imprinting and the warm inner glow. Curr. Biol..

[71] Haig, D. in *Social Behaviour: Genes*, *Ecology and Evolution* (eds T. Szekely, A.J. Moore, & J. Komdeur) 107-109 (Cambridge University Press, 2010).

[72] Arnold W (1990). The evolution of marmot sociality: II. Costs and benefits of joint hibernation. Behav. Ecol. Sociobiol..

[73] Clutton-Brock TH, Albon SD, Guinness FE (1989). Fitness costs of gestation and lactation in wild mammals. Nature.

[74] Koivula M, Koskela E, Mappes T, Oksanen TA (2003). Cost of reproduction in the wild: Manipulation of reproductive effort in the bank vole. Ecology.

[75] Vaanholt, L. M. et al. Limits to sustained energy intake. XXVII. Trade-offs between first and second litters in lactating mice support the ecological context hypothesis. *J. Exp. Biol.* **221**10.1242/jeb.170902 (2018). 10.1242/jeb.170902 29361590

[76] Konig, B. in *Cooperation in Primates and Humans*. *Mechanisms and Evolution*. (eds P.M. Kappeler & C.P. van Schaik) 191-205 (Springer-Verlag, 2006).

[77] Konig B (1989). Kin recognition and maternal care under restricted feeding in house mice (*Mus domesticus*). Ethology.

[78] Taborsky, M., Cant, M. A. & Komdeur, J. *The Evolution of Social Behaviour*. (Cambridge University Press, 2021).

[79] Marshall JA, Rowe JE (2003). Kin selection may inhibit the evolution of reciprocation. J. Theor. Biol..

[80] Quinones AE, van Doorn GS, Pen I, Weissing FJ, Taborsky M (2016). Negotiation and appeasement can be more effective drivers of sociality than kin selection. Philos. Trans. R. Soc. Lond. B Biol. Sci..

[81] Engelhardt SC, Weladji RB, Holand O, Roed KH, Nieminen M (2015). Evidence of reciprocal allonursing in reindeer, *Rangifer tarandus*. Ethology.

[82] Glonekova M, Brandlova K, Pluhacek J (2021). Further behavioural parameters support reciprocity and milk theft as explanations for giraffe allonursing. Sci. Rep..

[83] Ferrari M, Konig B (2017). No evidence for punishment in communally nursing female house mice (*Mus musculus domesticus*). PLoS One.

[84] Bateman N (1957). Some physiological aspects of lactation in mice. J. Agric. Sci..

[85] Downing PA, Griffin AS, Cornwallis CK (2020). The benefits of help in cooperative birds: Nonexistent or difficult to detect?. Am. Nat..

[86] Cockburn A (2008). Can we measure the benefits of help in cooperatively breeding birds: the case of superb fairy-wrens *Malurus cyaneus*?. J. Anim. Ecol..

[87] Meade J, Nam KB, Beckerman AP, Hatchwell BJ (2010). Consequences of ‘load-lightening’ for future indirect fitness gains by helpers in a cooperatively breeding bird. J. Anim. Ecol..

[88] Hager, R. & Jones, C. B. (Cambridge University Press, Cambridge, 2009).

[89] Cant, M. A., Nichols, H. J., Thompson, F. J. & Vitikainen, E. in *Cooperative Breeding in Vertebrates: Studies of Ecology, Evolution and Behavior* (eds W. D. Koenig & J. L. Dickinson) Ch. 18, 318-337 (Cambridge University Press, 2016).

[90] Thompson FJ (2016). Reproductive competition triggers mass eviction in cooperative banded mongooses. Proc. R. Soc. Ser. B.

[91] Thompson FJ (2017). Explaining negative kin discrimination in a cooperative mammal society. Proc. Natl Acad. Sci..

[92] Rood JP (1974). Banded mongoose males guard young. Nature.

[93] Hodge SJ (2009). Maternal weight, offspring competitive ability, and the evolution of communal breeding. Behav. Ecol..

[94] Roberts SA (2010). Darcin: a male pheromone that stimulates female memory and sexual attraction to an individual male’s odour. BMC Biol..

[95] Blaustein AR, O’Hara RK (1982). Kin recognition in *Rana cascadae* tadpoles: Maternal and paternal effects. Anim. Behav..

[96] Lize A, Cortesero AM, Atlan A, Poinsot D (2007). Kin recognition in *Aleochara bilineata* could support the kinship theory of genomic imprinting. Genetics.

[97] Gomez-Baena G (2019). Molecular complexity of the major urinary protein system of the Norway rat, *Rattus norvegicus*. Sci. Rep..

[98] MacLean B (2010). Skyline: an open source document editor for creating and analyzing targeted proteomics experiments. Bioinformatics.

[99] Team, R. C. R: A language and environment for statistical computing. *R Foundation for Statistical Computing* (2015).

[100] Bates D, Maechler M, Bolker B, Walker S (2014). lme4: Linear mixed-effects models using Eigen and S4. J. Stat. Softw..

[101] Perez-Riverol Y (2019). The PRIDE database and related tools and resources in 2019: improving support for quantification data. Nucleic Acids Res.

