# Supplementary material for: Cryptic kin discrimination during communal lactation in mice favours cooperation between relatives
Source: Commun Biol. 2023 Jul 15;6:734. doi: 10.1038/s42003-023-05115-3 (PMC10349843; doi:10.1038/s42003-023-05115-3)
Supplement: Supplementary file 1 — Supplementary Information [file 42003_2023_5115_MOESM1_ESM.pdf]

Supplementary Information

Cryptic kin discrimination during communal rearing in mice favours cooperation between relatives

Jonathan P. Green, Catarina Franco, Amanda J. Davidson, Vicki Lee, Paula Stockley, Robert J. Beynon & Jane L. Hurst

Supplementary Table 1. Relatedness between mothers does not influence the weight of pups reared in communal nests

Supplementary Table 2. Individual female milk investment, determined by food intake[#], depends on communal litter size not own litter size

Supplementary Fig. 1. Protein labelling and peptide to peptide consistency in calculation of relative isotope abundance (RIA).

Supplementary Fig. 2. Consistency of relative maternal investment calculation across proteins.

Supplementary Fig. 3. Correct partition of diets between co-housed females.

Supplementary Fig. 4. Relative investment gained by each pup from each female in communal nests of sisters (a) or unrelated females (b).

Supplementary Fig. 5. Kinship reduces energy required to rear pups in communal nests.

Supplementary Fig. 6. Tradeoff between time spent in the nest and time spent foraging by pairs of communally nursing females.

Supplementary Note 1. Calculation of relative investment (containing Supplementary Figures 7-9).

Supplementary Note 2. Distributions of residuals from statistical models (containing Supplementary Figures 10 – 17).

Supplementary Software 1. Relative investment calculator (separate excel file)

Supplementary Data 1. Investment data per pup and source data behind graphs in the paper (separate excel file)

Supplementary Table 1. Relatedness between mothers does not influence the weight of pups reared in communal nests [#]

Effect†	Partial η^2	F	df	$p^{‡}$
**Total weight of pups at day 14**				
Communal litter size	0.895	93.27	1,11	**< 0.0001**
Age of 2nd born litter	0.445	8.82	1,11	**0.013**
Mean weight of mothers prior to breeding	0.409	7.62	1,11	**0.019**
Relatedness between mothers	0.002	0.017	1,11	0.90
**Mean weight per pup at day 14**				
Communal litter size	0.474	9.91	1,11	**0.009**
Age of 2nd born litter	0.367	6.39	1,11	**0.028**
Mean weight of mothers prior to breeding	0.480	10.16	1,11	**0.009**
Relatedness between mothers	0.013	0.15	1,11	0.71

[#] Pup weight measured when first born pups reached 14 days old .

† Analysis of covariance, n = 16 nests (8 pairs of sisters, 8 pairs of unrelated females), data shown in Fig. 3 and Supplementary Data 1.

‡ Values in bolded text are statistically significant ($p < 0.05$).

Supplementary Table 2. Individual female milk investment, determined by food intake[#], depends on communal litter size not own litter size

Effect†	Fixed effect	χ^2	df	*p*[‡]
All pups				
Communal litter size	2.79 ± 0.81	10.32	1	**0.001**
Own litter size	-1.31 ± 1.26	1.05	1	0.30
Relatedness between females	-10.71 ± 3.13	9.36	1	**0.0022**

[#] Mixed-effects model of food eaten by female over the labelling period (g), including nest as a random effect. Data are for n=32 females in 8 sister and 8 unrelated female pairs (16 nests in total), shown in Fig. 5 and Supplementary Data 1.
[‡] Values in bolded text are statistically significant ($p < 0.05$).

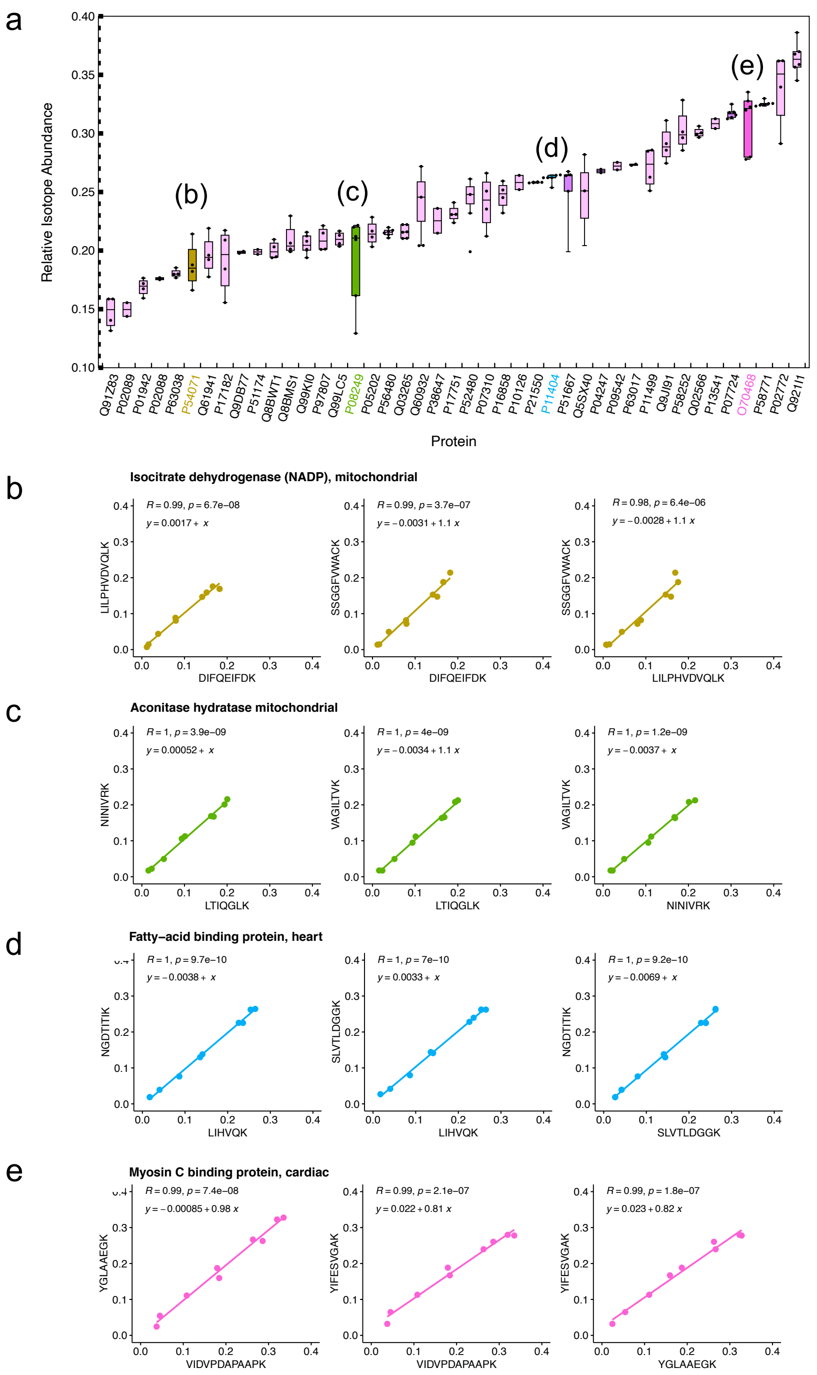

Supplementary Fig. 1. Protein labelling and peptide to peptide consistency in calculation of relative isotope abundance (RIA). Female mice with pups were labelled over 6 days and, at different times, pups were taken and heart tissues excised and analysed by bottom-up proteomics (n=9 pups). (a) The extent of labelling (RIA, ranging from zero [no labelling] to 0.5 [full labelling]) varied between proteins, depending on the rate of protein turnover and the degree of pool expansion of each protein (data from two pups taken after the full 6 day period of labelling in two pups). The RIA values are full and interquartile ranges across multiple peptides per protein with whiskers to full range. (b-e) Four proteins (marked b-e in panel a) examined more closely across all pups taken at different time points to assess the consistency in RIA between different peptides from the same protein (points show individual pup data). In all instances, the peptides were very highly correlated (r>0.98 in all cases), confirming confidence in using a single peptide to calculate the relative contribution of two mothers in subsequent experiments.

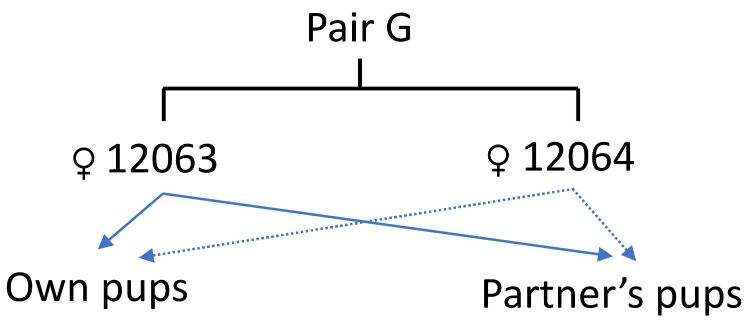

Percentage contribution from female 12063

	Protein			
Pup	Aco	FABP	IDH	MyBPC
G1_37293	52	53	51	50
G2_37294	52	53	50	51
G4_37296	50	51		51
G9_37301	54	59	53	52

	Protein			
Pup	Aco	FABP	IDH	MyBPC
G10_37302	50	49	53	52
G11_37303	52	51	57	54
G3_37295	53	54	57	53
G5_37297	52	55	57	54
G6_37298	54	52	57	54
G7_37299	49	50	48	49
G8_37300	53	55	58	55

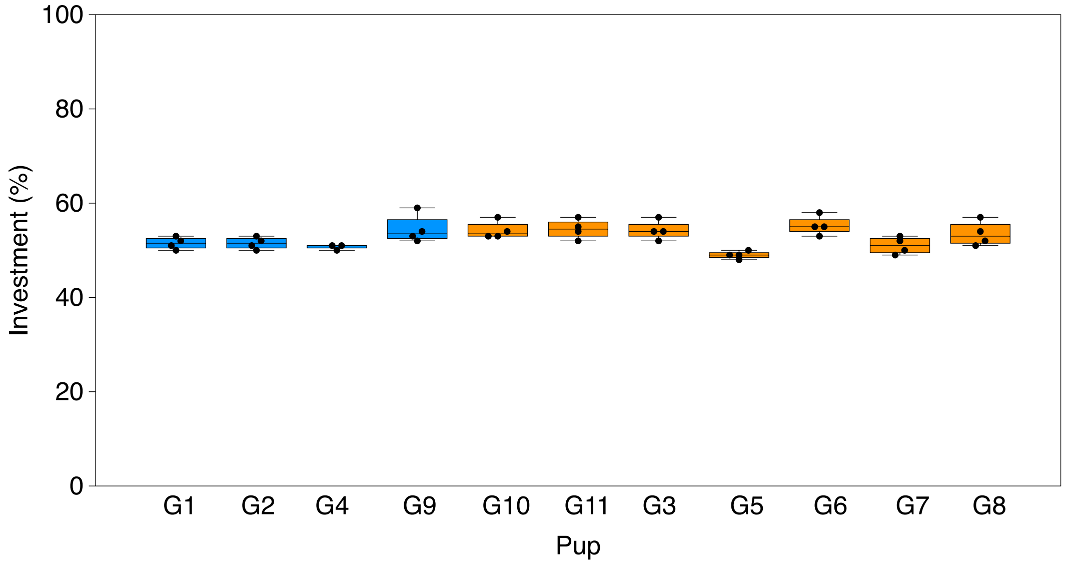

Supplementary Fig. 2. Consistency of relative maternal investment calculation across proteins. Illustrated for pups in one communal nest (G), the relative investment that pups gained from female 12063 (n=4 own pups [blue in boxplot], n=7 partner pups [orange in boxplot]) was calculated based on the labelling of four different proteins (Aco: aconitate dehydratase, FABP: fatty acid binding protein, IDH: isocitrate dehydrogenase and MyBPC: myosin binding protein C). Boxplot shows median, interquartile boxes with whiskers to full range and dots show individual proteins. The relative maternal investment calculated for each pup from different proteins is remarkably similar, despite differences in the extent of labelling between proteins (see Supplementary Fig. 1).

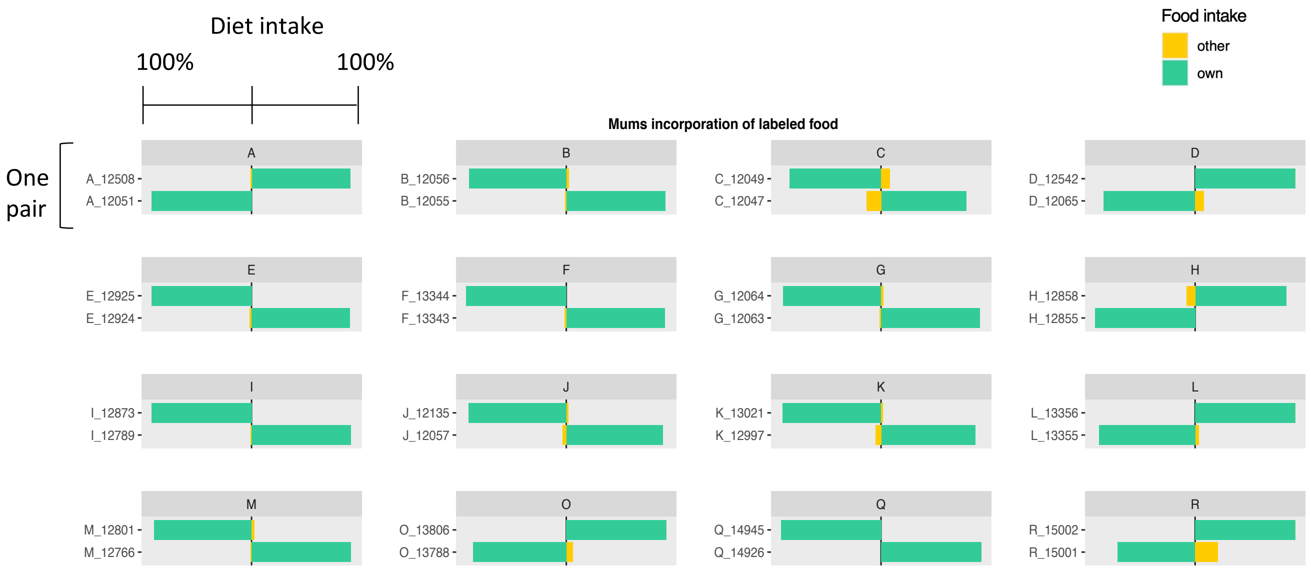

Supplementary Fig. 3. Correct partition of diets between co-housed females. Females in communally-nursing pairs (A through R) were each provided with RFID-controlled access to differently labelled diets. To assess the extent to which females consumed the incorrect diet (for example, by picking up food crumbs left by the partner female), urine samples were taken at the end of each experiment and analysed by proteomics. The labelling pattern for serum albumin in urine was used to assess the degree of incorrect labelling (shaded in yellow), compared to the degree of labelling with the correct diet (shaded in green) for each pair of females (top bar and bottom bar within each panel). The effectiveness of the diet partition system is evident, diet contamination averaging only 3.2%. This slight contamination was taken into account when calculating the relative investment received by pups from each mother (see Supplementary Note 1).

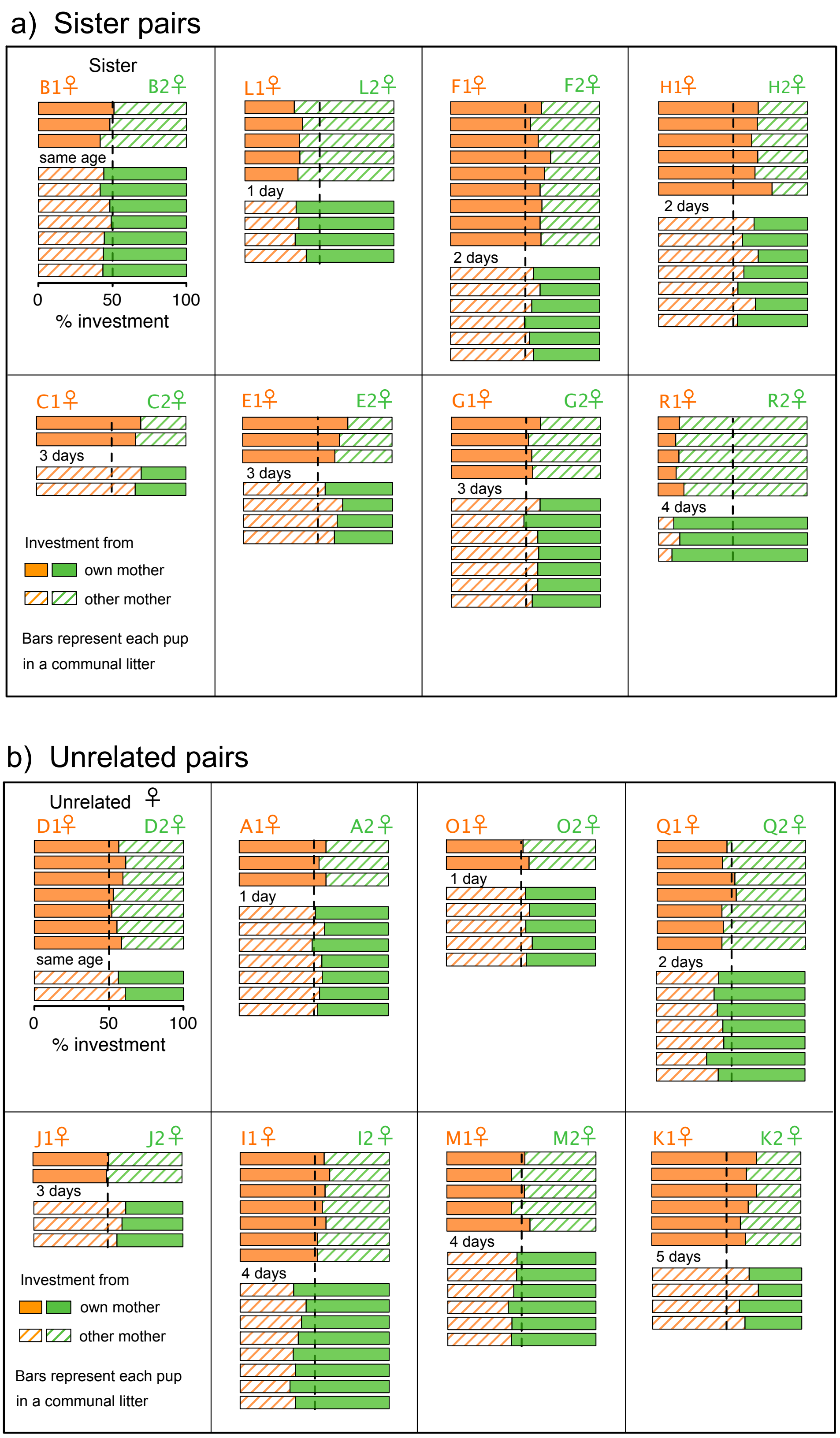

Supplementary Fig. 4. Relative investment gained by each pup from each female in communal nests of sisters (a) or unrelated females (b). Bars indicate the proportion of investment received from own mother (solid) or partner mother (hatched) for each pup in the combined nest. Vertical dashed lines marks equal investment from both females. Litter born first (female 1, orange) shown above litter born second (female 2, green) with age difference in between. If litters were born on the same day (pairs B & D), birth order was unknown and both litters were treated as first born in analyses.

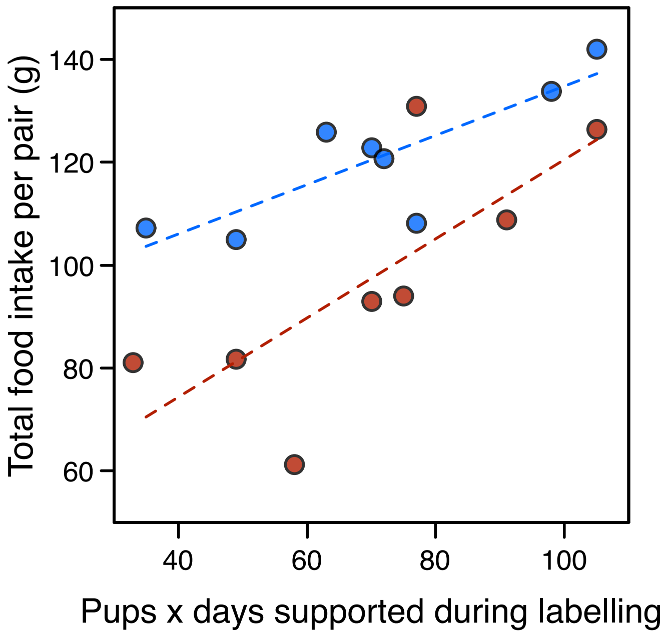

Supplementary Fig. 5. Kinship reduces energy required to rear pups in communal nests. To correct for the small number of pups that disappeared during the 7d milk labelling period, we calculated the total number of pups x days fed over this period for each communal nest to match to the total food intake by both mothers over the same period. This made very little difference compared to using the communal litter size measured at day 14 (see Fig. 3d). Analysis of covariance showed that the total food eaten by both mothers increased with the total number of pup days as expected ($F_{1,11}$ = 33.53, p < 0.001), but sisters required less food than unrelated pairs ($F_{1,11}$ = 9.30, p = 0.011). Regression lines for sister (red) and unrelated pairs (blue) shown as dashed lines.

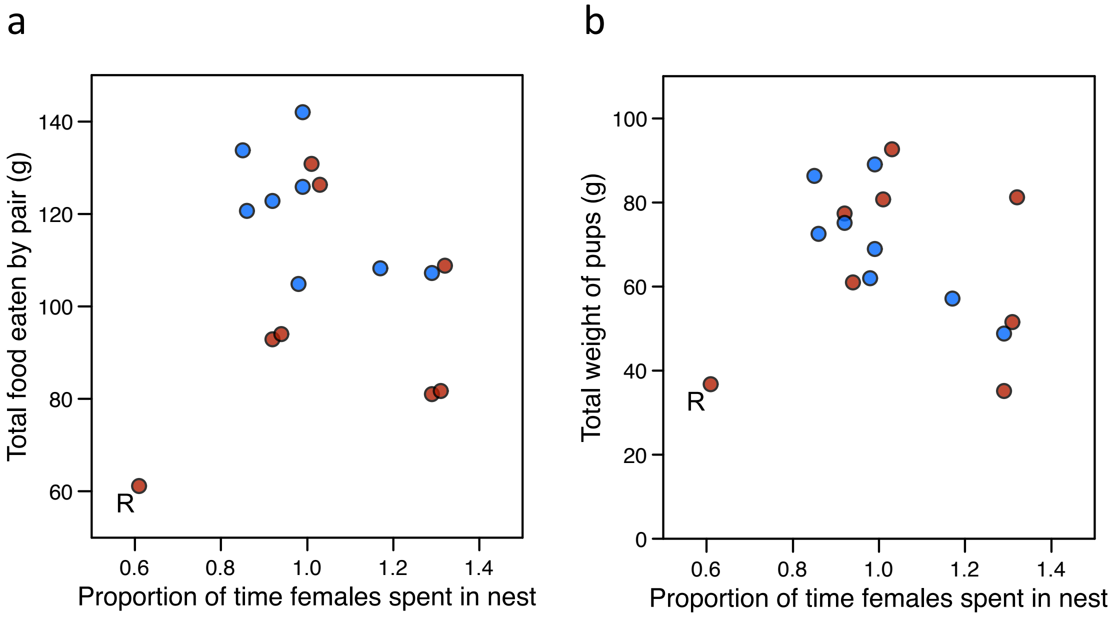

Supplementary Fig. 6. Tradeoff between time spent in the nest and time spent foraging by pairs of communally nursing females. The proportion of time that each mother spent in the nest was summed to give the total proportion of female time in the nest (potentially ranging 0-2). One pair (R) was an outlier that spent much less time in the nest than other pairs, despite spending little time foraging when out of the nest (**a**); the total weight of pups achieved by this pair was particularly low (**b**). Excluding pair R, there was a tradeoff between time spent with pups and time spent foraging (**a**: r = -0.53, p = 0.04), resulting in a negative relationship between time in the nest and total weight of pups achieved (**b**: r = -0.57, p = 0.03). Blue circles: unrelated pairs (n=8), red circles: sister pairs (n=8). Although unrelated females did not avoid spending time together in the nest, they spent more time on their own with pups than sisters did (sisters: 43.4 ± 2.3%, unrelated: 52.8 ± 1.5%; $F_{1,14}$ = 11.72, p = 0.005), reflecting the increased foraging time required by unrelated females.

Correction for isotopomer profiles

The experiment uses two isotopes d_4 lysine and d_9 lysine, both of which are administered at an abundance of approximately 0.5. Thus, there is also a significant signal from unlabelled peptide d_0. Because the monoisotopic (all ^{12}C) unlabelled peptide mass d_0 is 4 Da separated from the d_4 peptide and the d_4 peptide is 5 Da from the d_9 monoisotopic mass, we also made a minor correction to compensate for this spillover. This is peptide specific. For the peptide used in this analysis (NGDTITIK, derived from heart FABP), we derived the isotope profile using the MS-Isotope package in Protein Prospector (http://prospector.ucsf.edu Supplementary Figure 7).

Peptide with D0 lysine

Distribution Type: Peptide Sequence
Sequence: NGDTITIK
Parent Charge: 2
Intensity: 1
Profile Type: Gaussian
Resolution: 10000

Elemental Composition: C36 H65 N10 O14
Monoisotopic M/Z: 431.23745
Total Abundance: 100.00%

Isotope Number	m/z	Percent Total	Percent Maximum
0	431.2375	61.82	100
1	431.7389	28.11	45.47
2	432.2402	8.01	12.96
3	432.7414	1.71	2.76
4	433.2427	0.3	0.48
5	433.7439	0.04	0.07
6	434.2451	0.01	0.01
7	434.7463	0	0
8	435.2479	0	0

Peptide with D4 lysine

Distribution Type: Elemental Composition
Elemental Formula: C36 H61 N10 O14 D4
Parent Charge: 2
Intensity: 1
Profile Type: Gaussian
Resolution: 10000

Elemental Composition: C36 H61 N10 O14 D4
Monoisotopic M/Z: 433.25000
Total Abundance: 100.00%

Isotope Number	m/z	Percent Total	Percent Maximum
0	433.25	61.86	100
1	433.7515	28.09	45.41
2	434.2527	8	12.94
3	434.754	1.7	2.75
4	435.2552	0.3	0.48
5	435.7564	0.04	0.07
6	436.2576	0.01	0.01
7	436.7589	0	0
8	437.2605	0	0

Peptide with D9 lysine

Distribution Type: Elemental Composition
Elemental Formula: C36 H56 N10 O14 D9
Parent Charge: 2
Intensity: 1
Profile Type: Gaussian
Resolution: 10000

Elemental Composition: C36 H56 N10 O14 D9
Monoisotopic M/Z: 435.76570
Total Abundance: 100.00%

Isotope Number	m/z	Percent Total	Percent Maximum
0	435.7657	61.9	100
1	436.26715	28.07	45.34
2	436.76842	7.99	12.9
3	437.26968	1.7	2.74
4	437.7709	0.3	0.48
5	438.27211	0.04	0.07
6	438.77331	0.01	0.01
7	439.27456	0	0
8	439.77614	0	0

m/z	%	%	%
M0	61.82		
M1	28.11		
M2	8.01		
M3	1.71		
M4	0.3	61.86	
M5	0.04	28.09	
M6	0.01	8	
M7	0	1.7	
M8	0	0.3	
M9		0.04	61.9
M10		0.01	28.07
M11		0	7.99
M12		0	1.7
M13			0.3
M14			0.04
M15			0.01
M16			0
M17			0

Supplementary Figure 7: MS Isotope height distribution

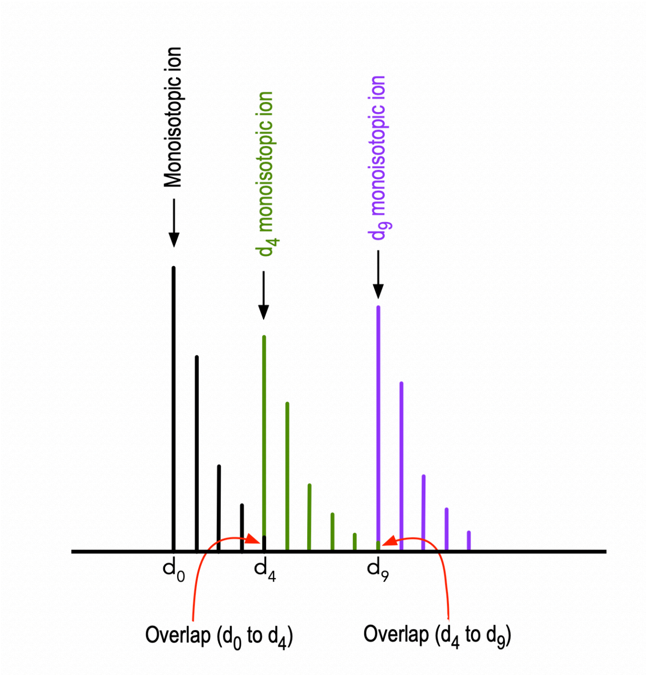

Supplementary Figure 8: Overlap of isotopomer profiles

To correct for the slight spillover from the overlap between the $d0$, $d4$ and $d9$ isotopologues of lysine

(Supplementary Figure 8), we corrected the intensities as follows. The three m/z peaks of interest, and their intensities, are at M_0, the monoisotopic unlabelled peak, (d_0, intensity $M0$), $M_0 + 4Da$ (the $d4$ labelled peptide, intensity $M4$) and $M_0 + 9Da$ (the d_9 labelled peptide, intensity $M9$). The intensities of these three ions are related thus:

$$Int_{d4,A} = M4 - 0.0049 \cdot M0 \tag{1}$$

$$Int_{d9,A} = M9 - 0.007 \cdot Int_{d4,A} \tag{2}$$

Overall analytical considerations

One mother is labelled with d_4 lysine, the other, in the same cage, is labelled with d_9 lysine. In both instances the diet is labelled to an isotopic enrichment of 50%, to maintain palatability of the diet. It follows that the milk from each mother contains unlabelled d_0 lysine but the milk from the two mothers is distinguishable by the presence or absence of d_4 lysine or d_9 lysine in addition.

Thus, the only two isotopic peptide ions of interest are those for peptides labelled with d_4 lysine or d_9 lysine. All further calculations are based on a 'local' Relative Isotopic Abundance (RIA) which reflects the relative enrichment of d_4 lysine or d_9 lysine, expressed as a fraction of the summed abundance of d_4 lysine and d_9 lysine. The 'local' RIA thus ranges from 0 to 1 for both mothers, and 0 to 1 for the pups.

Calculation of maternal input

One mother is labelled with d_4 lysine, the other with d_9 lysine. Although the feeders are designed to restrict access of each mouse to its 'own' diet, there was a possibility of some ingestion of the 'wrong' diet from, for example, discarded crumbs. For this calculation, we used the true ion intensities, corrected for isotopic spillover (see above). This diet 'cross-contamination' was slight, averaging 96.8% correct diet, 3.2% incorrect diet across all females.

For the mother A intended to receive d_4, the local RIA is calculated as:

$$RIA_{d4,A} = \frac{Int_{d4,A}}{(Int_{d4,A} + Int_{d9,B})} \tag{3}$$

$$RIA_{d9,A} = \frac{Int_{d9,A}}{(Int_{d4,A} + Int_{d9,B})} \tag{4}$$

and for the mother B intended to receive d_9,

$$RIA_{d4,B} = \frac{Int_{d4,B}}{(Int_{d4,A} + Int_{d9,B})} \tag{5}$$

$$RIA_{d9,B} = \frac{Int_{d9,B}}{(Int_{d4,A} + Int_{d9,B})} \tag{6}$$

where the subscripts '$d4$' and '$d9$', refer to isotopes and 'A' and 'B' refer to mothers respectively.

Calculation of relative investment in pups

Peptides from each pup tissue (P) will yield a signal for d_4 and d_9 that reflect the fractional input of mother A (F) and mother B $(1 - F)$. The RIA for each isotope is calculated as:

$$RIA_{d4,P} = \frac{Int_{d4,P}}{(Int_{d4,P} + Int_{d9,P})} \tag{7}$$

and

$$RIA_{d9,P} = \frac{Int_{d9,P}}{(Int_{d4,P} + Int_{d9,P})} \tag{8}$$

The RIA values for the two isotopes can be expressed in terms of the RIA of each mother:

$$RIA_{d4,P} = RIA_{d4,A} \cdot F + RIA_{d4,B} \cdot (1 - F) \tag{9}$$

and

$$RIA_{d9,P} = RIA_{d94,A} \cdot F + RIA_{d9,B} \cdot (1 - F) \tag{10}$$

These are a pair of simultaneous equations, and the value of F is derived using the Solver function in Excel (Supplementary Figure 9, Supplementary Software 1). The RIA values for each mother and the pup, for each isotope, are entered, and the Solver returns the value of F, expressed as a percentage. For this study, the RIA values for each isotopomer were the average of all mothers.

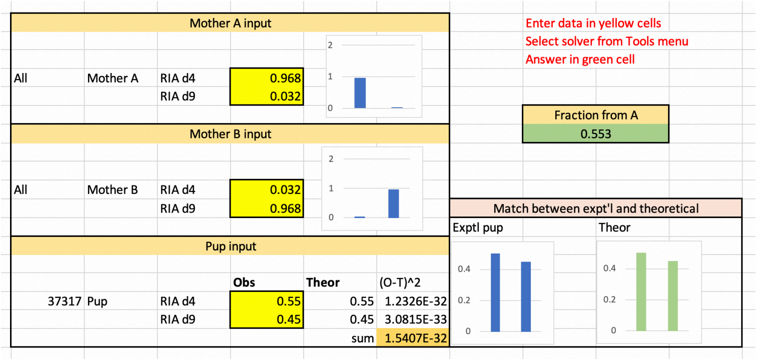

Supplementary Figure 9: Excel Solver template for calculation of relative investment.

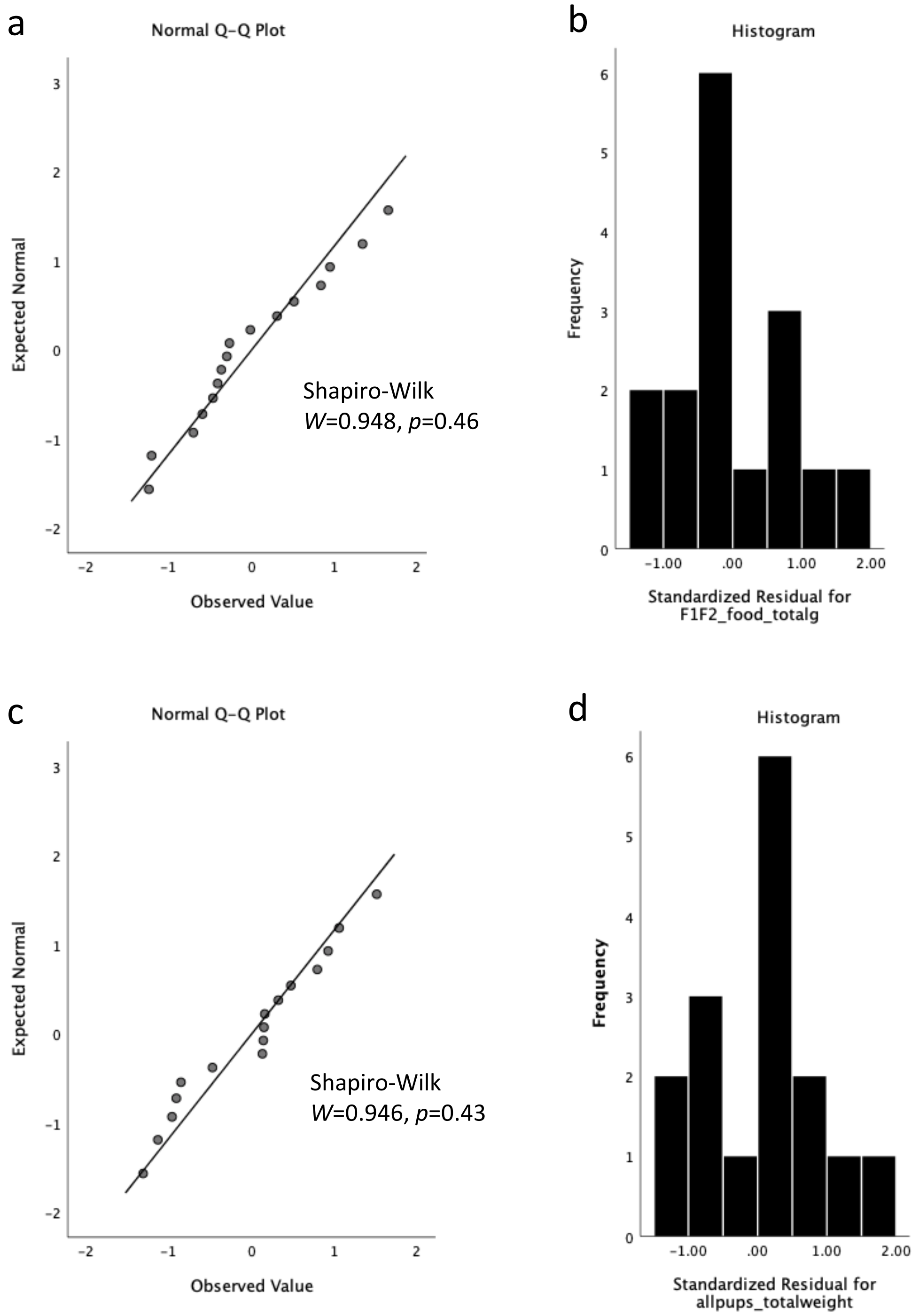

Supplementary Figure 10. Relatedness between mothers influences the relationship between total food intake, communal litter size and pup weight. Distribution of residuals from models shown in Table 1 investigating factors influencing total food eaten by pair (a,b), and total weight of pups at day 14 (c,d). Normal Q-Q plots (a,c), frequency histograms (b,d) and Shapiro Wilk tests confirmed the good fit of data to each model.

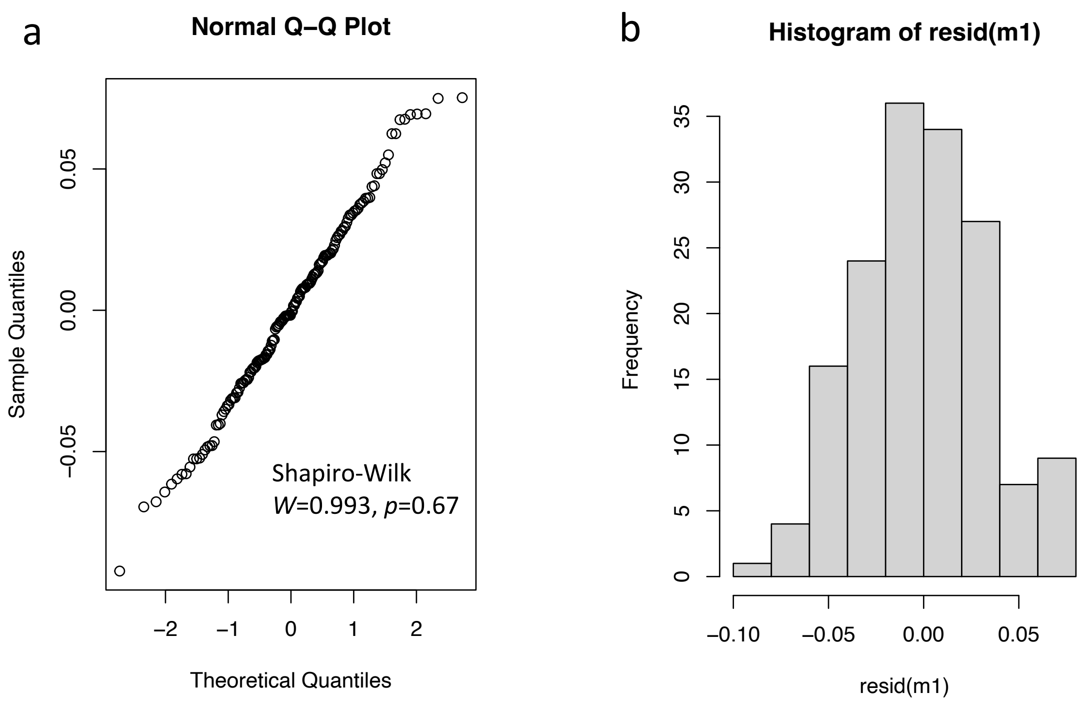

Supplementary Figure 11. Proportion of investment gained by pups from focal female in each pair. Distribution of residuals from model in Table 2. Normal Q-Q plot (a), frequency histogram (b) and Shapiro Wilk test confirmed the good fit of data to the model.

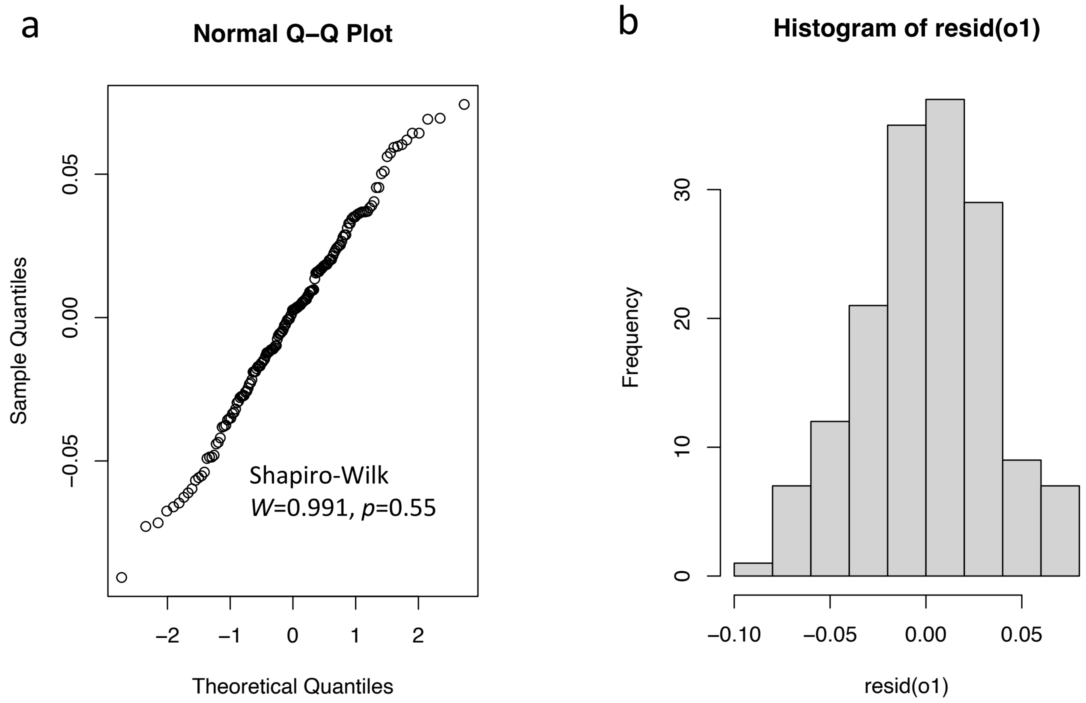

Supplementary Figure 12. Effect of communal litter size and litter birth order on relative investment gained from partner mother by all pups. Distribution of residuals from model in Table 3 investigating relative investment gained by all pups. Normal Q-Q plot (a), frequency histogram (b) and Shapiro Wilk test confirmed the good fit of data to the model.

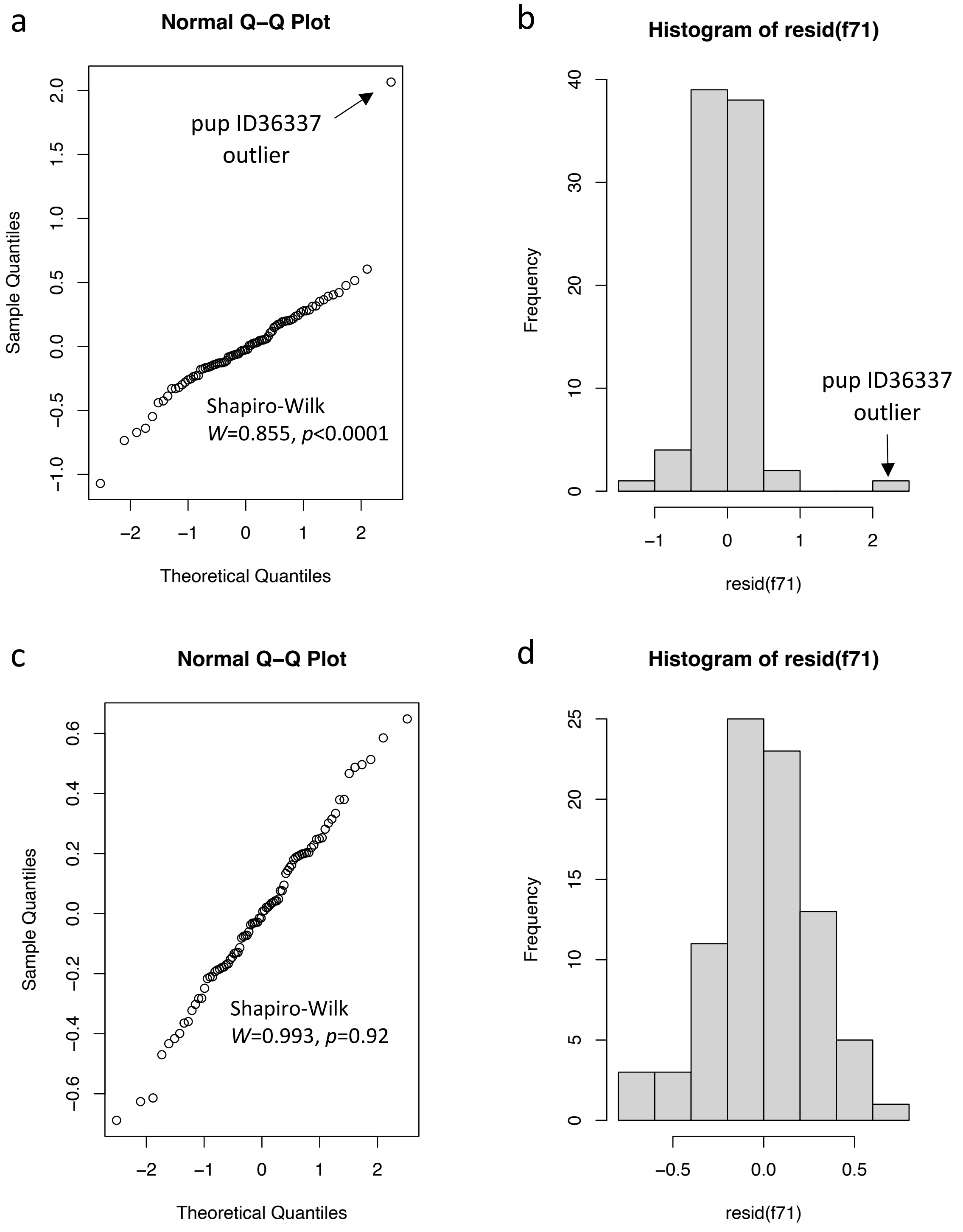

Supplementary Figure 13. Factors influencing the body weight of first-born litter pups by day 14. Normal Q-Q plot (a) and frequency histogram (b) of residuals from an initial model that included all first-born litter pups and factors shown in Table 4 revealed a clear outlier (pup ID36337). As this pup weight was abnormally large compared to other pups in the same litter (10.6g versus 7.16 +/- 0.14g) and no note was made of such an obvious difference at the time, the data point recorded was most likely an error. Removal of this data point from the model (see Table 4, first-born pups) resulted in good fit of the data to the model confirmed by a Normal Q-Q plot (c), frequency histogram (d) and a Shapiro Wilk test. Factors showing a significant effect in Table 4 were also significant ($p < 0.05$) before removal of pup ID36337 from the model.

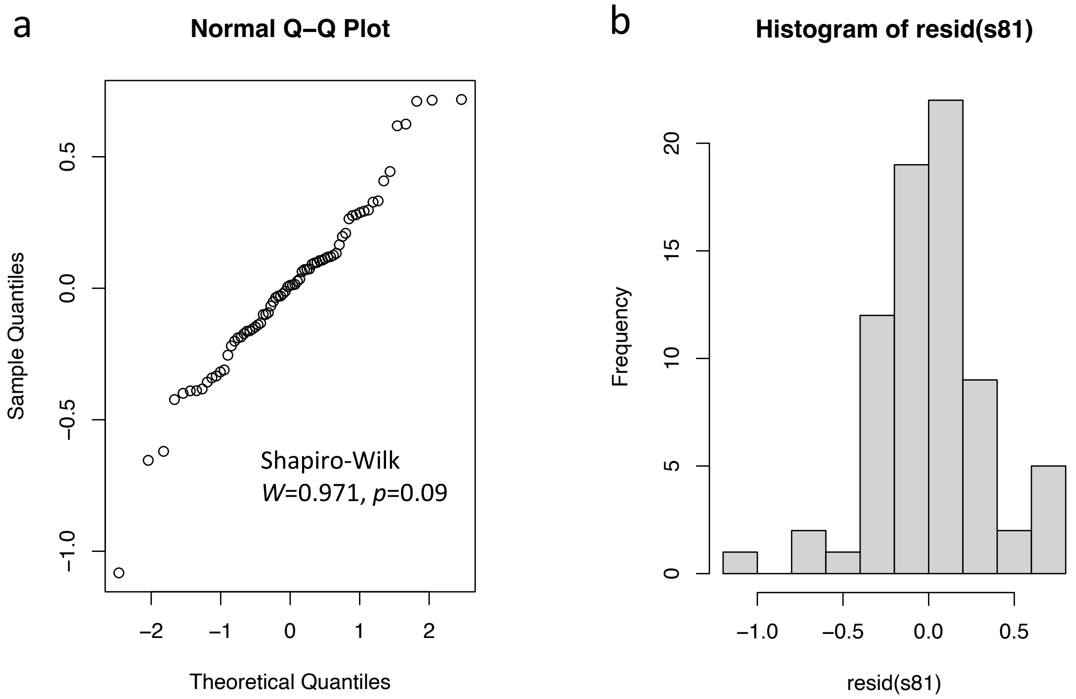

Supplementary Figure 14. Factors influencing the body weight of second-born litter pups by day 14. Distribution of residuals from model for pups in second-born litters shown in Table 4. Normal Q-Q plot (a), frequency histogram (b) and Shapiro Wilk test confirmed the good fit of data to the model.

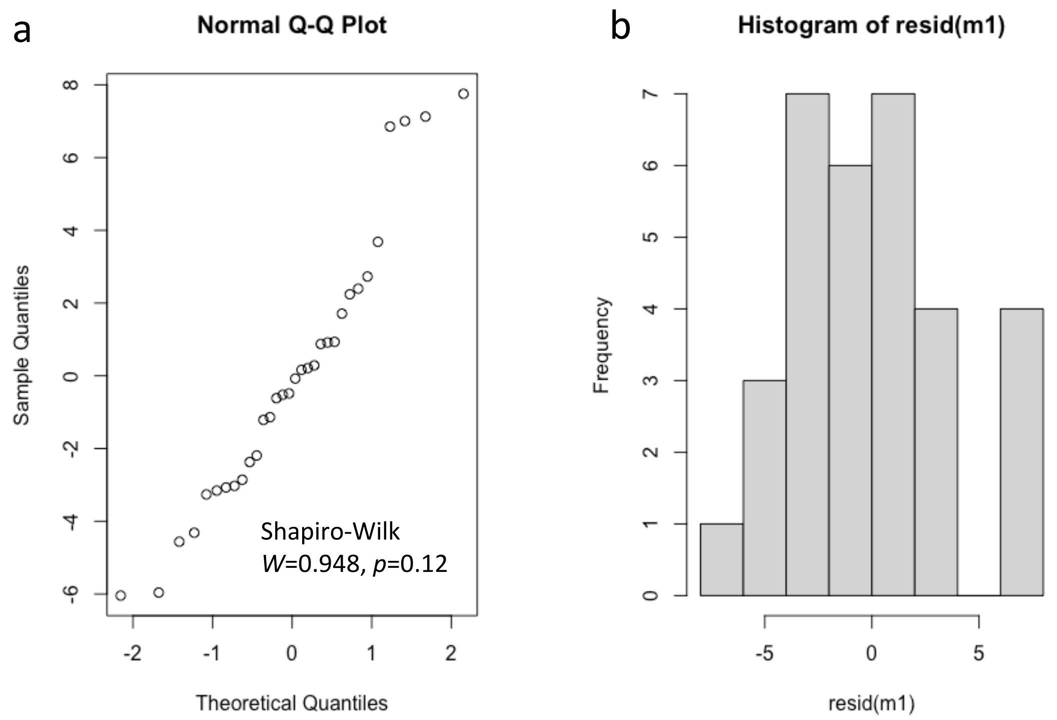

Supplementary Figure 15. Individual female energetic cost per own offspring raised is lower when rearing pups communally with a sister compared to an unrelated partner, whether females have more or fewer pups than their partner. Distribution of residuals from model shown in Table 5. Normal Q-Q plot (a), frequency histogram (b) and Shapiro Wilk test confirmed the good fit of data to the model.

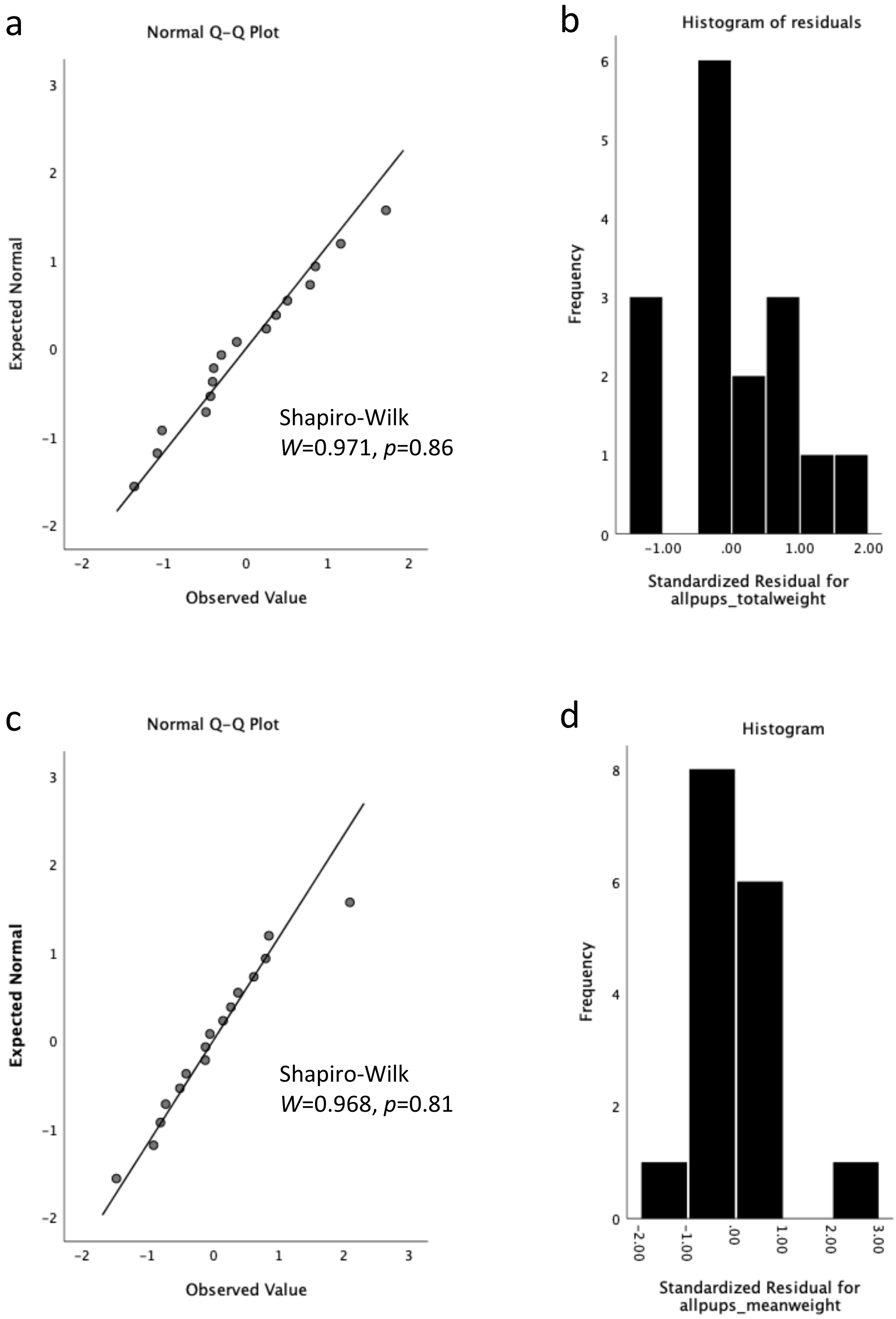

Supplementary Figure 16. Relatedness between mothers does not influence the weight of pups reared in communal nests. Distribution of residuals from models shown in Supplementary Table 1 investigating factors influencing total weight of pups at day 14 (a,b), and mean weight per pup at day 14 (c,d). Normal Q-Q plots (a,c), frequency histograms (b,d) and Shapiro Wilk tests confirmed the good fit of data to each model.

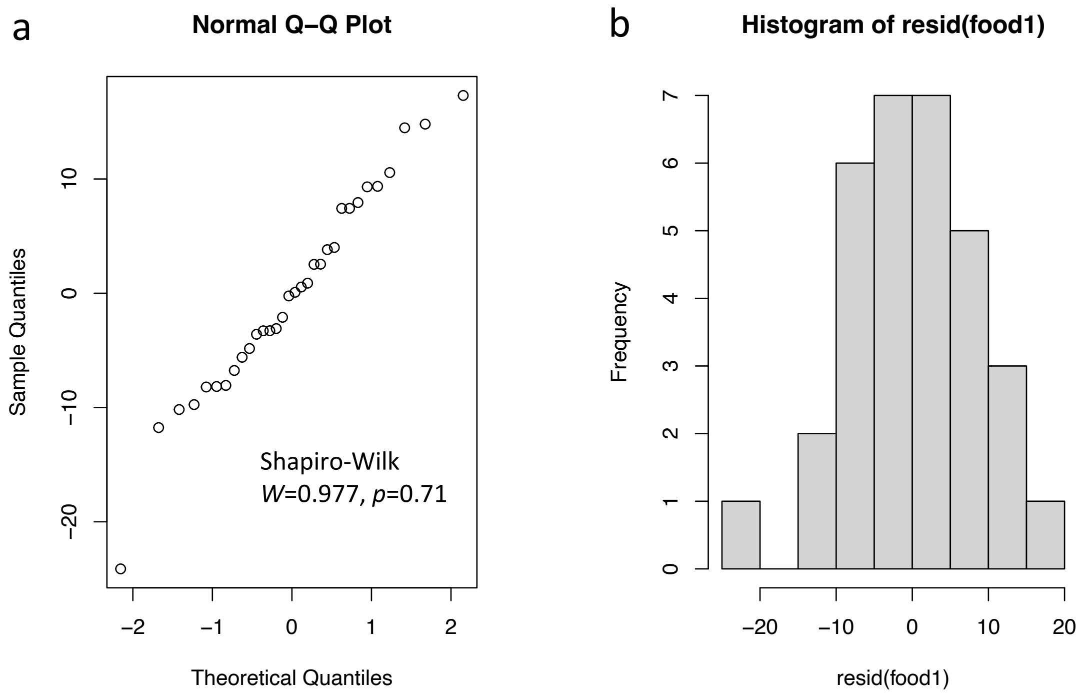

Supplementary Figure 17. Individual female milk investment, determined by food intake, depends on communal litter size not own litter size. Distribution of residuals from model shown in Supplementary Table 2. Normal Q-Q plot (a), frequency histogram (b) and Shapiro Wilk test confirmed the good fit of data to the model.
