# Supplementary material for: Cryptic kin discrimination during communal lactation in mice favours cooperation between relatives
Source: Commun Biol. 2023 Jul 15;6:734. doi: 10.1038/s42003-023-05115-3 (PMC10349843; doi:10.1038/s42003-023-05115-3)
Supplement: Supplementary file 2 — Description of Additional Supplementary Files [file 42003_2023_5115_MOESM2_ESM.pdf]

Description of Additional Supplementary Files

File name: Supplementary Software 1

Description: Relative investment calculator (separate excel file)

File name: Supplementary Data 1

Description: Investment data per pup and source data behind graphs in the paper
