# Supplementary material for: Cryptic kin discrimination during communal lactation in mice favours cooperation between relatives
Source: Commun Biol. 2023 Jul 15;6:734. doi: 10.1038/s42003-023-05115-3 (PMC10349843; doi:10.1038/s42003-023-05115-3)
Supplement: Supplementary file 5 — Reporting Summary [file 42003_2023_5115_MOESM5_ESM.pdf]

nature portfolio

Corresponding author(s): Prof Jane Hurst

Last updated by author(s): Sep 7, 2022

Reporting Summary

Nature Portfolio wishes to improve the reproducibility of the work that we publish. This form provides structure for consistency and transparency in reporting. For further information on Nature Portfolio policies, see our Editorial Policies and the Editorial Policy Checklist.

Statistics

For all statistical analyses, confirm that the following items are present in the figure legend, table legend, main text, or Methods section.

n/a	Confirmed	
☐	☒	The exact sample size (*n*) for each experimental group/condition, given as a discrete number and unit of measurement
☐	☒	A statement on whether measurements were taken from distinct samples or whether the same sample was measured repeatedly
☐	☒	The statistical test(s) used AND whether they are one- or two-sided<br>*Only common tests should be described solely by name; describe more complex techniques in the Methods section.*
☐	☒	A description of all covariates tested
☐	☒	A description of any assumptions or corrections, such as tests of normality and adjustment for multiple comparisons
☐	☒	A full description of the statistical parameters including central tendency (e.g. means) or other basic estimates (e.g. regression coefficient) AND variation (e.g. standard deviation) or associated estimates of uncertainty (e.g. confidence intervals)
☐	☒	For null hypothesis testing, the test statistic (e.g. *F*, *t*, *r*) with confidence intervals, effect sizes, degrees of freedom and *P* value noted<br>*Give P values as exact values whenever suitable.*
☒	☐	For Bayesian analysis, information on the choice of priors and Markov chain Monte Carlo settings
☒	☐	For hierarchical and complex designs, identification of the appropriate level for tests and full reporting of outcomes
☐	☒	Estimates of effect sizes (e.g. Cohen's *d*, Pearson's *r*), indicating how they were calculated

Our web collection on statistics for biologists contains articles on many of the points above.

Software and code

Policy information about availability of computer code

Data collection	Calculations and custom code used to calculate relative investment received by pups from each female is given in Note S1 and Equation S1 submitted with the manuscript.
Data analysis	Analyses were performed in R (v. 3.6.2) [82] or SPSS (IBM version 27) as stated in Data Analysis section.

For manuscripts utilizing custom algorithms or software that are central to the research but not yet described in published literature, software must be made available to editors and reviewers. We strongly encourage code deposition in a community repository (e.g. GitHub). See the Nature Portfolio guidelines for submitting code & software for further information.

Data

Policy information about availability of data

All manuscripts must include a data availability statement. This statement should provide the following information, where applicable:

- Accession codes, unique identifiers, or web links for publicly available datasets
- A description of any restrictions on data availability
- For clinical datasets or third party data, please ensure that the statement adheres to our policy

The mass spectrometry proteomics data for the main study (relative investment) have been deposited to the ProteomeXchange Consortium via the PRIDE [84] partner repository with the dataset identifier PXD01 9578. In addition, the milk pilot study proteomics data have been deposited at the same site with identifier PXD019586. All other data needed to evaluate the conclusions in the paper are provided in Data S1.

Human research participants

Policy information about studies involving human research participants and Sex and Gender in Research.

Reporting on sex and gender	*Use the terms sex (biological attribute) and gender (shaped by social and cultural circumstances) carefully in order to avoid confusing both terms. Indicate if findings apply to only one sex or gender; describe whether sex and gender were considered in study design whether sex and/or gender was determined based on self-reporting or assigned and methods used. Provide in the source data disaggregated sex and gender data where this information has been collected, and consent has been obtained for sharing of individual-level data; provide overall numbers in this Reporting Summary. Please state if this information has not been collected. Report sex- and gender-based analyses where performed, justify reasons for lack of sex- and gender-based analysis.*
Population characteristics	*Describe the covariate-relevant population characteristics of the human research participants (e.g. age, genotypic information, past and current diagnosis and treatment categories). If you filled out the behavioural & social sciences study design questions and have nothing to add here, write "See above."*
Recruitment	*Describe how participants were recruited. Outline any potential self-selection bias or other biases that may be present and how these are likely to impact results.*
Ethics oversight	*Identify the organization(s) that approved the study protocol.*

Note that full information on the approval of the study protocol must also be provided in the manuscript.

Field-specific reporting

Please select the one below that is the best fit for your research. If you are not sure, read the appropriate sections before making your selection.

☒ Life sciences ☐ Behavioural & social sciences ☐ Ecological, evolutionary & environmental sciences

For a reference copy of the document with all sections, see nature.com/documents/nr-reporting-summary-flat.pdf

Life sciences study design

All studies must disclose on these points even when the disclosure is negative.

Sample size	A minimum sample size of eight pairs of littermate sisters and eight pairs of unrelated but familiar and socially compatible females that reared their offspring communally was set at the start of the study. This allowed for natural variation in the timing of births within pairs, which was set to a maximum age difference of up to 5 days between first-born and second-born litters to match communal nests typical of free-ranging wild mice. All pups from each of these communal nests were sampled, resulting in 44 first born and 33 second born pups in sister nests, and 41 first born and 40 second born pups in unrelated female nests, representing 8 genetically distinct communal nests of each type. Both nest and mother are taken into account in statistical analyses as appropriate. All other females bred that did not meet the required criteria were retained in the colony along with their offspring as stock mice.
Data exclusions	All pairs meeting the requirements of the study and their pups were included in analyses. One small communal litter of sister pups was excluded from data analysis and replaced with another sister pair after analysis of pup tissues revealed that all pups were fed by only one of the sister partners, so failed to meet the requirement of communal rearing. Two unrelated pairs were replaced after analysis of female urine confirmed that a technical feeder fault led to cross-contamination of labelled diets between the mothers. When examining factors influencing pup body weight, one male pup was a clear outlier and was excluded from this particular analysis, as detailed in the paper, where we checked that this made no difference to the conclusions from the analysis.
Replication	Each replicate pair of partner mice in our study consisted of genetically unique outbred wild house mice. Each female was mated to a different genetically distinct male, such that pups represented a broad range of genotypes. Tissues and weights from all pups within each litter were assessed, providing replication within each litter. Our isotopic tracer approach underwent extensive development to ensure the reliability and replicability of measurement across pups, different tissues, proteins and peptides. Measures replicated across pups within the same litter showed strong similarity as evident from results reported in the paper.
Randomization	Sisters and unrelated females were from the same outbred breeding colony of wild-stock house mice and selected according to limited availability of females of matched age and prior breeding experience. Pairs of females were excluded if they showed signs of social incompatibility (any overt aggression) as female house mice only select familiar compatible nestmates for communal breeding partners. To be included in the study of communal rearing, both females within a pair had to give birth within a restricted time period, keep at least one own pup to the end of the experiment (day 14 from the first pups born), and pups had to be fed by both partner females. Details of pairs that failed to meet these criteria are given in the Methods subsection 'Subjects', with analysis showing that a similar proportion of females in sister and unrelated pairs failed to rear offspring. As these breeding criteria were determined by the animals, we checked that this resulted in no significant differences in the weights or weight differences between partners, the age difference between litters within the same communal nest or the mean age of pups at day 14 of the experiment between sister and unrelated pairs. Females within pairs were randomly assigned to the two different labels that were incorporated in their otherwise identical laboratory diet.
Blinding	Experimenters and other animal care staff were not blinded to the identity of mice used in the experiments. This would have been very

Blinding	difficult to achieve given the requirement for careful matching of animals of limited availability and the need to stagger replicates over time. However, each tissue and urine sample was given a unique 5 digit code prior to mass spectrometric analysis and relative investment gained by each pup was calculated using a standardised equation. For the majority of data collected, most of the team collecting the data were blind to the specific hypotheses being tested during data collection.

Reporting for specific materials, systems and methods

We require information from authors about some types of materials, experimental systems and methods used in many studies. Here, indicate whether each material, system or method listed is relevant to your study. If you are not sure if a list item applies to your research, read the appropriate section before selecting a response.

Materials & experimental systems

n/a	Involved in the study
☒	☐ Antibodies
☒	☐ Eukaryotic cell lines
☒	☐ Palaeontology and archaeology
☐	☒ Animals and other organisms
☒	☐ Clinical data
☒	☐ Dual use research of concern

Methods

n/a	Involved in the study
☒	☐ ChIP-seq
☒	☐ Flow cytometry
☒	☐ MRI-based neuroimaging

Animals and other research organisms

Policy information about studies involving animals; ARRIVE guidelines recommended for reporting animal research, and Sex and Gender in Research

Laboratory animals	Subjects were captive-bred female and male Mus musculus domesticus derived from ancestors captured from six populations in the northwest of England UK and maintained in an outbred colony. Parents were aged 4-14 months when mated and offspring were aged up to 14 days.
Wild animals	*Provide details on animals observed in or captured in the field; report species and age where possible. Describe how animals were caught and transported and what happened to captive animals after the study (if killed, explain why and describe method; if released, say where and when) OR state that the study did not involve wild animals.*
Reporting on sex	The question addressed concerning lactation investment relates only to females. Data for pups of both sexes are reported.
Field-collected samples	*For laboratory work with field-collected samples, describe all relevant parameters such as housing, maintenance, temperature, photoperiod and end-of-experiment protocol OR state that the study did not involve samples collected from the field.*
Ethics oversight	All animal care protocols were in accordance with the University of Liverpool Animal Welfare Committee requirements, with EU Directive 2010/63/EU and UK Home Office guidelines for animal care. Tissue samples from live animals were obtained under UK Home Office licence under the Animals in Scientific Procedures Act 1986, according to best practice guidelines. The University of Liverpool Animal Welfare Committee approved the work.

Note that full information on the approval of the study protocol must also be provided in the manuscript.
